# Memorization of Strain-Induced Moiré Patterns in Vertical van der Waals Materials

**DOI:** 10.1021/acsami.4c22462

**Published:** 2025-03-04

**Authors:** Aditya Dey, Nazmul Hasan, Stephen M. Wu, Hesam Askari

**Affiliations:** † Department of Mechanical Engineering, University of Rochester, Rochester, New York 14611, United States; ‡ Department of Electrical and Computer Engineering, University of Rochester, Rochester, New York 14620, United States; § Department of Physics and Astronomy, University of Rochester, Rochester, New York 14611, United States

**Keywords:** strain-induced moiré patterns, strain memorization, machine-learned interatomic potential, atomistic simulations, interface mechanics

## Abstract

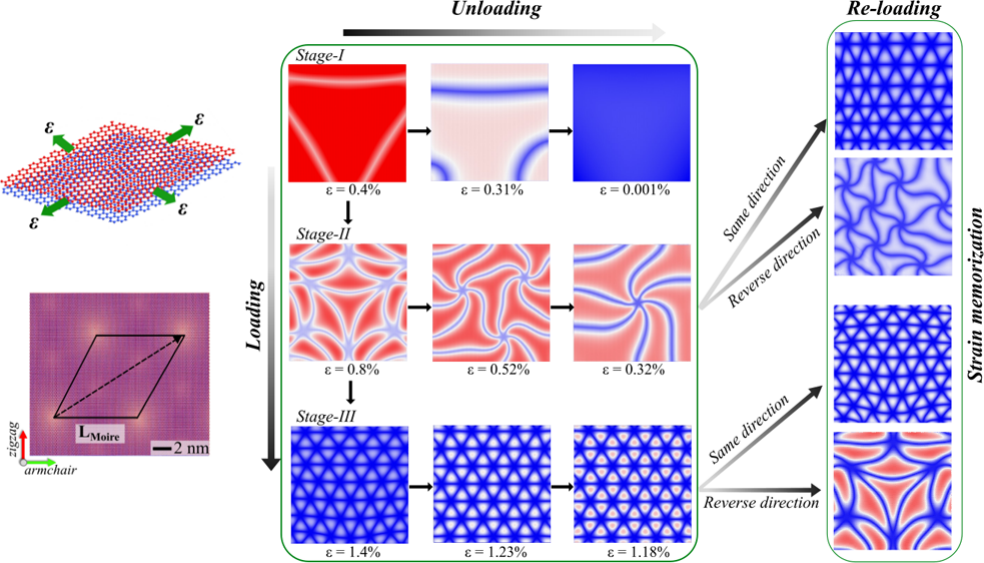

Twisting layers in van der Waals (vdW) materials have traditionally produced moiré patterns but often suffer from alignment issues and nonuniformity due to the sensitivity of twist angles. Applying strain alone can also generate these patterns, eliminating the need for interlayer rotation and enabling controlled, reproducible moiré formation. We present the mechanistic principles governing the evolution of strain-induced moiré patterns in vertically stacked graphene through atomistic simulations. By analyzing local strain distribution, we identify a three-stage interlayer slippage process responsible for pattern formation. Our analyses reveal that these triangular moiré domains are stable and retained upon unloading, ensuring consistent and reproducible pattern formation even after strain removal. Additionally, we demonstrate that this strain history can be utilized to reapply load in a step-by-step process to achieve uniform moiré domains without requiring higher strain magnitudes. This approach provides a robust mechanism for designing wafer-scale quantum materials with uniform and reproducible moiré superlattices.

## 1 Introduction

Manipulation of atomic configurations in stacked van der Waals (vdW) materials offers a dynamic method to tune their optical and electronic properties. For example, inducing relative interlayer rotation by twisting two or more vdW layers has traditionally been used to form moiré patterns^1−4^ that are known to produce unconventional behaviors such as strongly correlated states,^5−7^ superconductivity,^8−10^ and topological phases^11−13^ in various families of 2D materials, including twisted bilayer graphene and twisted transition metal dichalcogenides (TMDs).^14−18^ These structures are primarily obtained by stacking single layers of 2D materials with a rotation or by folding a single layer onto itself. However, this process is prone to alignment issues, wrinkle formation, and inaccuracies in maintaining the twist angle, leading to challenges in the reproducibility and repeatability of experiments involving twisted 2D materials.^19−23^ This is particularly concerning because many exotic properties are only achieved within a narrow twist angle range, typically between 0.9 and 1.2°, where even small deviations can significantly affect the reproducibility of the experiments.^19^

An alternate way to manipulate interlayer atomic configurations is through heterostraining, which applies inequivalent strain to the layers of a vertical vdW material. This process introduces a lattice mismatch between the strained layers, generating moiré patterns without the need for twist. Hence, this method has the potential to overcome many challenges arising from the lack of control in the application of the twist angle. The use of process-induced straining technique as demonstrated in our previous works^24−27^ provides utmost control over strain-induced lattice mismatches, effectively eliminating a major source of nonuniformity and irreproducibility by avoiding twist. In this work, we present the fundamental mechanisms responsible for the formation of strain-induced moiré patterns and reveal how strain is memorized within graphene as a representative vertical vdW material. The existence of strain memorization implies that the periodicity and shape of strain-induced patterns can be preserved once the load is removed. This effect is essential for the stability and conservation of moiré domains postformation and upon removal of the load. Furthermore, we show that strain memorization incorporates a directionality component that allows recovering a partially formed spiral pattern to fully formed triangular patterns in graphene. This effect enables the creation of local subdomains in a step-by-step manner, without necessitating the application of high-strain magnitudes in a single procedure to form stable triangular domains.

**Figure 1:**
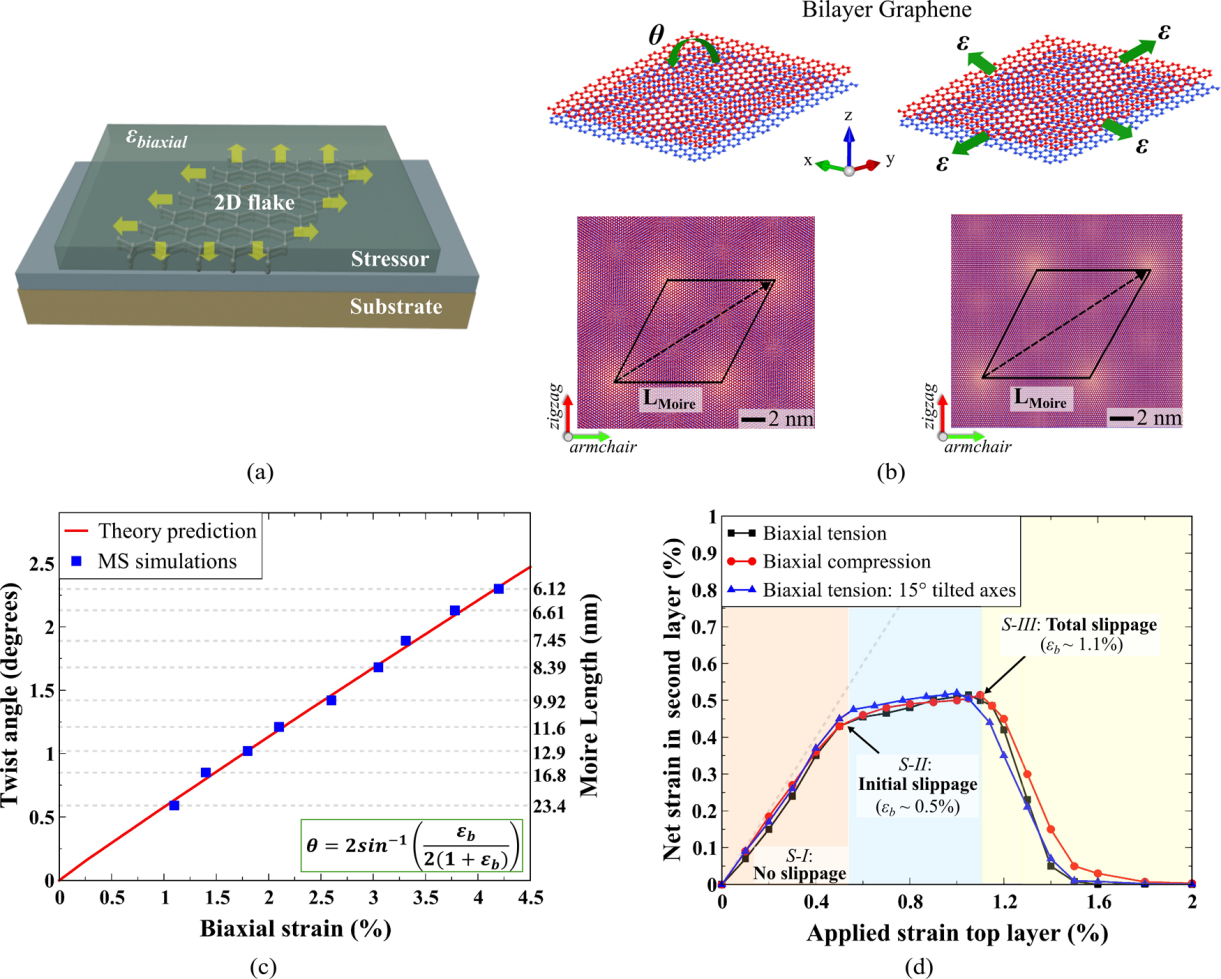
Atomistic model and strain transfer behavior. (a) Schematic of an experimental setup, where a 2D flake is biaxially strained using a thin film stressor deposited on a substrate. (b) Illustration of moiré patterns in graphene formed by two different mechanical stimuli: twist and strain. A close atomic-scale snapshot of a real-space graphene flake under the respective mechanical stimuli and the corresponding moiré superlattice generated is shown (depicted by the black rhombus). The snapshots reveal that moiré patterns formed by these techniques are structurally similar in length but configurationally different in atomic arrangements. The lengths of strain-induced moiré superlattices can be compared mathematically with those generated by applying interlayer rotation (θ), establishing a direct relationship between the twist angle and biaxial strain magnitude, as shown in panel (c). The plot demonstrates the comparison of this relationship, aligning well between theoretical estimates and molecular statics (MS). (d) Average strain in the layer beneath the top layer as a function of the applied strain in the top layer. Complete strain transfer occurs initially in stage-I up to 0.5% load. The gray dotted line represents the expected strain in the second layer for perfect strain transfer. Strain propagation is reduced due to the first slip after 0.5% (stage-II) and continues until 1.1% (stage-III), beyond which total slippage occurs with negligible strain transfer. This three-stage loading behavior is consistent for biaxial tension and compression along perfect zigzag and armchair directions, as well as along varied loading axes (15° tilted).

The method of moiré engineering through the application of heterostrain can be effectively implemented using a process-induced straining technique that involves controlled strain application in vdW materials. Originally developed to improve the performance of silicon-based transistors, this strained-Si technology has been widely used in the industry since the 90 nm technology node to improve electron and hole mobility in transistor channels.^28^ The technique involves the deposition of a stressed capping layer, also referred to as a stressor, consisting of evaporated stressed thin films. The choice of the stressor material determines the tensile or compressive state of stress, while its thickness controls the stress magnitude applied to the 2D films. For example, compressive thin films such as MgO and SiO_2_ expand to relieve stress and induce tensile strain, while tensile films such as MgF_2_ contract to alleviate stress, thus inducing compressive strain. This allows for a deterministic design of strain directionality and alignment with crystal axes, as demonstrated in the schematic of Figure 1a. Other straining techniques like substrate bending, bulge testing, MEMS-tensile tests, etc., might not be very effective in achieving such controlled strain directionality and magnitude necessary for obtaining strain-induced moire patterns. This highlights the critical importance of the process-induced strain method in precisely regulating strain in stacked vdW materials. Moreover, this method is also nondamaging and offers robust time and temperature stability as compared to other methods.^26,29^ Our group has demonstrated the exceptional degree of such control in our previous works with multilayered 2D materials, driving applications such as phase change, memristor behavior, and stacking order changes.^24,26,27,29−31^

In this work, we use an atomistic simulation framework to examine the formation of moiré patterns at different loading conditions. Our framework models the experimental process-induced straining method, a simulation approach we have utilized in our previous works. By specifically modeling this experimental technique for strain-induced moiré pattern simulations, we aim to provide analyses that closely resemble the experimental process, making our findings readily applicable to experiments. Since strain can be removed after pattern formation, we study the stability of the patterns, the thresholds for their stability, the possibility of further evolution of partially or fully formed patterns, and the role of the strain history and direction on moiré pattern formation. We collectively refer to these phenomena as the strain memorization effect. To examine this effect, we use a setup consisting of three stacked layers of graphene in a Bernal stacking configuration without any twist, with the bottommost layer fixed. This setup mimics the stressor-based method, where the bottom layer is anchored to a substrate, and only the top layer is strained by the stressor. In our model, only the top two layers are freestanding and experience strain. The fixed layer does not interfere with interlayer strain mechanisms, as the strain transfer length scale in graphene is limited to two layers, as reported in our previous studies.^24,26^ We perform these simulations using the molecular statics (MS) method, developing a machine-learned interatomic potential (MLIP) trained on our density functional theory (DFT) data to define atomic interactions in the models. Our need for MLIP arises from the inability of traditional potentials to examine interlayer interactions and interface-driven mechanics that form and evolve moiré patterns.^30,32−38^ Using such a framework, this work reveals the evolution mechanisms and elucidates how we can design a step-by-step approach to achieve stable and uniform strain-induced moiré patterns.

## 2 Computational Methods

### 2.1 Machine-Learned Interatomic Potential (MLIP)

We first generate the training and testing data sets to construct the MLIP based on a deep neural network model, utilizing the DeepMD toolkit.^39,40^ Initial ab initio data are obtained through first-principles simulations using the Quantum Espresso package^41−45^ by incorporating various structures such as strained and unstrained stacked graphene, along with twisted moiré superlattice configurations (ranging from 21.79 to 6.1° twist angles).^31,37,46^ We construct these structures and apply minor perturbative atomic displacements from their original configurations using ab initio molecular dynamics (AIMD) to precisely capture the subtle lattice distortions induced by strain. This approach provides a robust data set essential for training the ML model to accurately predict the atomic configurations leading to moiré subdomains under heterostrain. The details of DFT calculations and training of MLIP with DFT data are included in the Supporting Information.

### 2.2 Molecular Statics (MS)

The developed DeepMD ML potential is then employed to conduct MS simulations using the LAMMPS package.^47^ We examine different vertically stacked graphene flake sizes ranging from 25 to 250 nm to elucidate the mechanisms behind strain-engineered moiré patterns across various flake dimensions. We apply a linear distribution of displacement to the top layer of graphene such that it remains under a uniform strain distribution (Figure 1a), an approach we used for our previous works as well.^29,30,48,49^ The specified displacement along both axes at each step corresponds to a net incremental 0.05% biaxial strain. The second layer can deform spatially in response to the applied strain. The overall simulation box and the whole system have free surface boundary conditions. Since we perform molecular statics (MS) simulations, the layer beneath the top strained layer minimizes its energy at each strain step, enabling a quasi-static response that accurately captures atomic reconfigurations, particularly those triggered by interlayer vdW interactions. A third fixed layer is added at the bottom to model a stressor-based experimental setup where the bottom-most layer has perfect adhesion to the substrate. The flake edges were passivated with hydrogen atoms to prevent edge effects that could distort the results. Additionally, we introduced a vacuum space of 40 Å along the out-of-plane direction to eliminate any potential interactions between periodic images. Further details about the MS simulations are available in the Supplementary Section.

## 3 Results and Discussion

### 3.1 Strain Localization and Mechanical Behavior at the Interface

We first apply biaxial strain to the top layer of Bernal stacked graphene, similar to the conditions that are present in stressed capping layer experiments. We have considered Bernal (AB) stacked graphene as it represents the most stable and naturally occurring stacking order during synthesis.^50^ Other stacking arrangements of graphene would result in similar lattice mismatches at the interface due to the evolving moire patterns. Previous studies have also reported that regardless of the initial stacking configuration, the resulting moiré subdomains would ultimately evolve to the different stacking arrangements (AB/BA, AA, SP) of stacked graphene.^51−53^ A representative strain-induced pattern from MS simulation is shown in Figure 1b and compared with a pattern obtained by only twist. Unlike uniaxial heterostrain that only produces straight-lined strain solitons,^30,54−56^ applying biaxial load uniformly induces lattice mismatch along the crystal axes, which ultimately results in formation of triangular moiré patterns.^57−59^ The length of moiré superlattice generated by biaxial heterostraining can be mathematically related to the twist angle θ using θ = 2 sin^–1^(ϵ_b_/(2 + 2ϵ_b_)), where ϵ_b_ is the net biaxial strain applied. This expression is derived by comparing the moiré superlattice lengths (*L*_m_) of that obtained by twist^53^: *L*_m_ = *a* sin(θ/2)/2 and that obtained by pure biaxial strain^57^: *L*_m_ = *a*(1 + ϵ_b_)/ϵ_b_, where *a* is the crystal lattice constant. MS simulations show an agreement in moiré length with the above equation as shown in Figure 1c. The figure shows that the length of the moiré patterns obtained by straining can be precisely related to those obtained by twisting but stacking configurations and stability of these patterns should not be expected to be identical. The reason is that one is created by a lattice stretching or compression and the other is obtained by inducing a rotation in the lattice.

**Figure 2:**
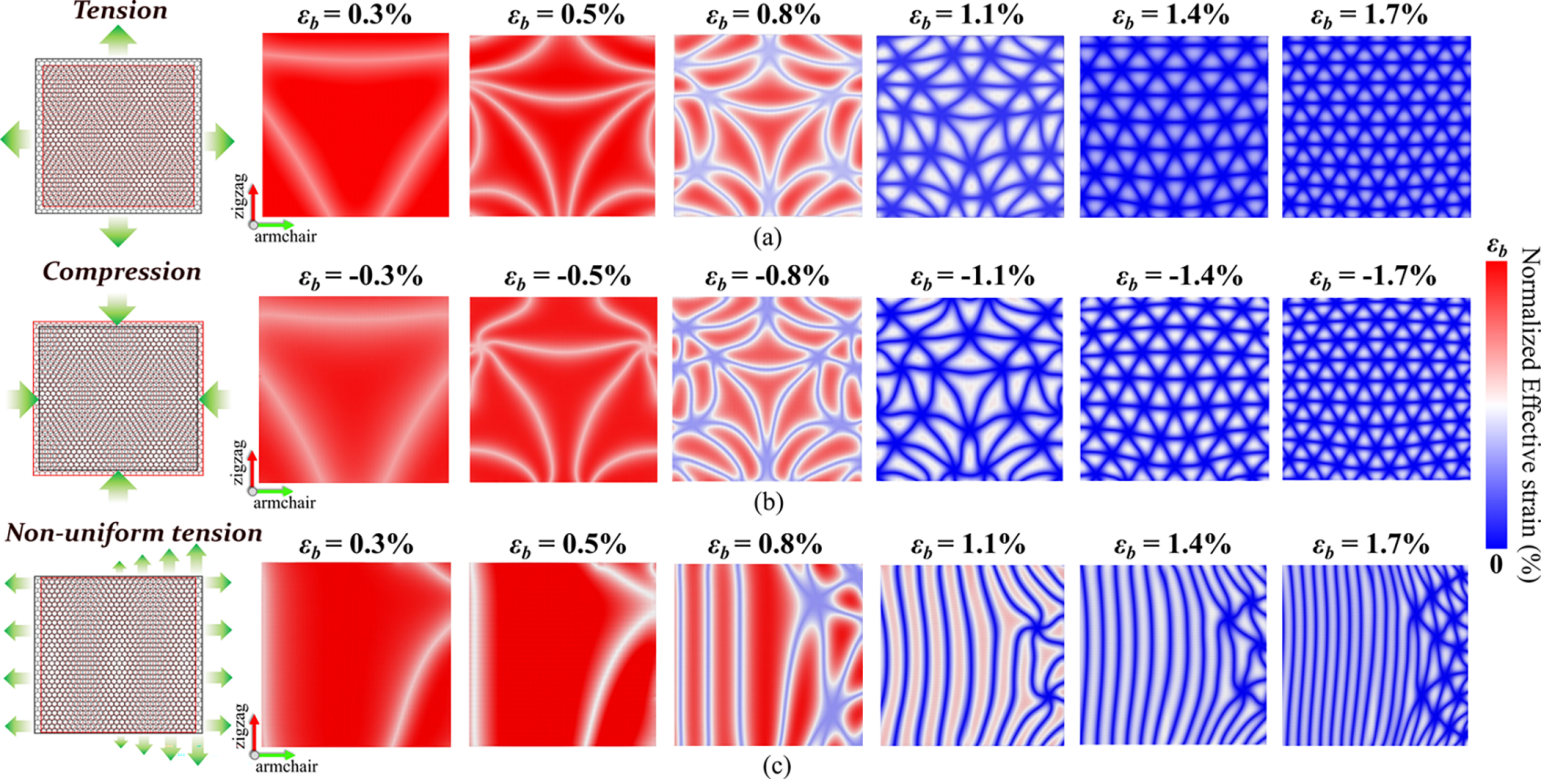
Strain localization and relaxation. Effective strain distribution contour plots of the second layer for 100 nm flake while applying (a) biaxial tension, (b) biaxial compression, and (c) nonuniform tension to the top layer. Each contour is calibrated using the displayed scale bar, with the upper limit representing the biaxial strain applied to the top layer (ϵ_b_) and the lower limit indicating zero strain. Disordered and distorted moiré-like subdomains are evident in stages I and II, becoming well-ordered upon complete slippage in stage-III. For nonuniform loading, uniform tension is applied along the armchair axis and a gradient tensile strain along the zigzag axis starting from the center of the flake. Straight strain solitons emerge after the first slip and further integrate into evolving moiré domains at higher strain. The strain distribution in stage-III shows the formation of fully developed moiré patterns resembling those obtained by interlayer twist.

The stressor deposited on a stacked vdW material exhibits strong adhesion to the top layer inducing strain in that layer, which is subsequently transferred to the subsequent layers. Our model assumes similar perfect adhesion, thus allowing only the top layer to be directly strained. The strain is then transferred to the stacked bottom layers during simulations via interaction forces defined by the MLIP. No adhesion is considered between the graphene layers, and the subsequent layer is strained solely by the strain transferred from the top layer through interlayer vdW interactions. As shown in Figure 1d, the strain transferred to the second layer compared to the applied strain reveals three regions separated by two distinct slip events. The strain transferred to the second layer and the applied strain to the top layer (shown by the dotted line in Figure 1d) diverge early in the straining process but are comparable in the first stage up to strain magnitudes of about 0.5%. This indicates a nonlinear elastic behavior in stage-I as discussed later in this article. A major slippage event occurs around 0.5% which drastically changes the strain transfer ratio to the stacked layer beneath. This marks the beginning of stage-II. The interface transfers a considerably lower strain ratio to the stacked layer below until a second major slip event occurs around the applied strain of 1.1%. This eventually leads to negligible strain transfer in stage-III, which shows total slippage between the layers and full reconstruction of atoms to form local domains as shown in Figure 2. These domains evolve further with continued straining with negligible strain transfer to the second layer. This behavior is generally insensitive to the compressive or tensile mode of loading and to the alignment of loading axes to the film directions, as presented in Figure 1d. Importantly, this three-stage behavior is consistent across various flake lengths, as supported by the data presented in the supplements and Figures S3 and S4.

We present effective strain maps in the second layer beneath the top strained layer to examine the evolution of strain and localization patterns for a 100 nm flake in Figure 2. Other flake length maps are reported in the Supplements (Figure S2). The effective strain in 2D is calculated as  where ε_*xx*_, ε_*yy*_ are normal strain components and ε_*xy*_ represent shear strain. The strain maps are individually normalized to the applied strain (ϵ_b_) to improve the examination of fine local variations in strain. During stage-I, the top layer effectively transmits the strain to the second layer. This results in the emergence of channel-like regions shown in white around 0.3% applied strain where strain is locally reduced. These regions expand and merge progressively with continued strain and form seemingly irregular patterns. They give rise to areas of significantly reduced strain shown by blue regions in the strain contour map as the flake transitions into stage-II where initial slippage occurs. Further straining results in evolution of these patterns which ultimately reshape to full triangular domains in stage-III.

### 3.2 Evolution of Moiré Patterns and Subdomains

The results shown in Figure 2a,b represent ideal biaxial loading conditions. To illustrate the effect of strain heterogeneity, we examined a specific case where strain is uniaxial in half of the flake and biaxial with a bias in the other half as shown in Figure 2c. The goal is to show that the resulting moiré pattern is closely related to the distribution of the level of control over the applied strain. Also, it is sometimes difficult to achieve a perfectly uniform biaxial strain field across both crystal axes.^29,60^ Even in well-controlled process-induced strain setups, variations in adhesion strength, stressor geometry, sample damages, and local strain relaxation mechanisms can lead to nonuniform strain distributions. It is important to understand how lattice mismatch evolves in cases where the strain deviates from a perfectly uniform biaxial load and whether the system can still develop triangular moiré patterns or if different structural motifs emerge. The effective strain distribution plots of this setup reveal that the biaxial load portion forms strain-relaxed regions until the first slippage at 0.5% strain, which is comparable to the same threshold observed in homogeneous biaxial loading (Figure 2a,b). We also observe additional straight soliton lines emerging in the uniaxially strained portion after the first slip as shown previously.^54,55^ The straight soliton regions continue to increase and the skewed-triangular moiré domains begin to form on the biaxial half. At higher strains beyond 1.1%, we see developing moiré patterns with triangular subdomains on the biaxial side of the flake. Interestingly, the interface mechanism and strain propagation to induce moiré patterns remain consistent across different loading conditions. These results show that the threshold for the two stages of slip in a mixed-mode loading is generally comparable to biaxial loading.

These strain localization maps in Figure 2 hint at changes from perfect Bernal stacking and creating AA and SP subdomains. It is well-known that the local domains and atomic positions of moiré patterns in twisted vdW systems undergo extensive reconfiguration to minimize the systems’ total energy. The interplay between in-plane elastic energy and interlayer vdW energy results in what is referred to as moiré reconstruction.^1,19^ This process involves the local subdomains of the structure adjusting their atomic configurations to maximize the formation of commensurate AB domains while simultaneously shrinking the incommensurate AA (circular domains) and SP (rectangular channels) domains for the case of graphene. This is driven by differences in interlayer spacing (ILS), with AA domains exhibiting the largest ILS (3.61 Å, followed by SP (3.46 Å), and AB domains exhibiting the smallest spacing (3.34 Å). As a result, we observe larger areas dominated by the most stable AB domains, followed by intermediate SP domains, and finally, the least stable AA circular domains. This has been observed and studied extensively in several studies,^1,25,61^ including our previous works.^27,53^ We observe a similar type of reconstruction and out-of-plane corrugation in subdomains with varying ILS for strain-induced moiré patterns, which reflects the behavior seen in twisted moirés. The illustration of such ILS variation is depicted in ILS contour plots (see Figure S9) for two representative structures, 1.4 and 1.7% tensile strained configurations. The contour maps clearly reveal a gradient of ILS, with the maximum occurring in AA regions, decreasing gradually in SP and AB domains.

**Figure 3:**
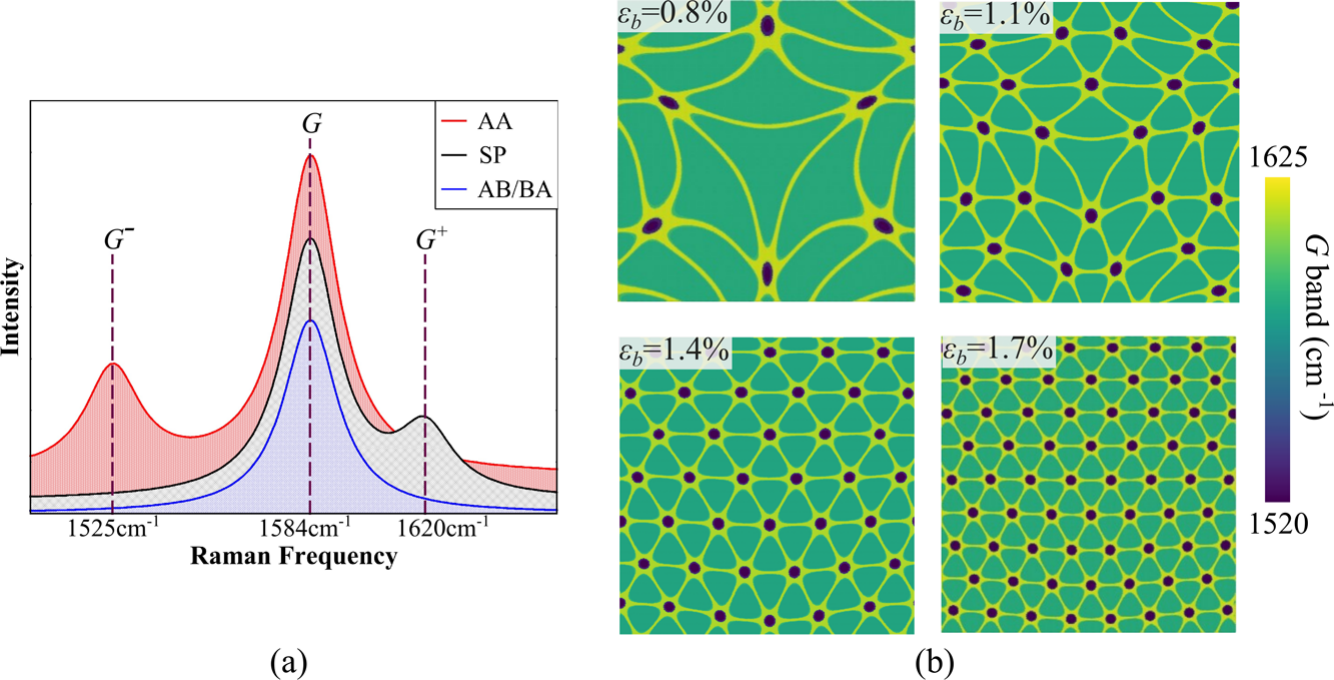
Subdomain stacking analysis through localized Raman. Local computational Raman is calculated via phonon dispersion plots using MLIP. The local atomic environment is identified and periodic unit lattices of each stacking type are extracted. Phonon spectra for all such lattices are automated, deriving optical phonon frequencies at the Γ point. The three known stacking types—AA, AB/BA, and SP—are distinguished by their marginally distinct G-band peak frequencies, as demonstrated in the (a) schematic Raman plot. AA and SP exhibit distinct peaks from the regular G-band peak, respectively, denoted as  and  peaks. These individual G-peaks are used to construct localized Raman contour plots for ϵ_b_ = 0.8, 1.1, 1.4, and 1.7% cases of *L*_flake_ = 100 nm, shown in panel (b).

The strain maps also suggest that commensurate and incommensurate domains emerge as a result of moiré reconstruction similar to what is obtained by twist. These domains are challenging to capture experimentally due to their small atomic resolution. In our previous work, we developed an approach to distinguish between stacking types using a combination of ILS and energy mapping.^53^ We expanded on this here by using Raman spectroscopy as a more easily detectable experimental measure to study the local subdomains. The atomic arrangements obtained by MS simulations can be used to create an atomic-scale Raman map to examine stacking configurations and to determine how they are related to the strain maps. We follow a procedure in which we identify 3 neighbors of each atom in our simulation environment and then extract a 4-atom periodic unit lattice to compute a localized Raman signature. We then automate the calculation of phonon spectra for all such lattices using the MLIP potential, deriving the optical phonon frequencies at the Γ point. The three known stacking types—AA, AB, and SP—can be distinguished by their distinct peak frequencies of the G-band (Figure 3a, as reported in previous studies.^62−66^ Stacking types of AA and SP exhibit distinct peaks from the regular G-band peak, denoted as  for AA and  for SP.^62,63^ Using these individual G-band frequencies, we construct localized Raman contour plots, assigning each atom to its respective G peak as derived from the phonon spectra (Figure 3b). Atoms in AA and SP stacking arrangements have two G-band wavenumbers:  for AA and  for SP, along with the common G peak. Atoms with AB arrangement show only one degenerate G-band peak. This approach allows for direct characterization of the AA, AB, and SP stacking regions within complex evolving moiré patterns across various strained configurations. The Raman maps in Figure 3 show that the strain localization maps shown in Figure 2 are directly related to known types of bilayer graphene stacking. Therefore, we show that the driving factor for strain localization is a change in the local stacking configuration.

It is important to note that our method can be generalized to create a variety of in-plane strain-induced moiré patterns, depending on how strain is applied. We have demonstrated the cases of uniform biaxial strain and an additional case of nonuniform gradient-based biaxial loading. The results reveal distinct atomic reconfiguration trends in response to such strain variations. These findings emphasize that deviating from equal, uniform biaxial loading results in a variety of distinct moiré configurations, extending beyond triangular domains. Any form of heterostrain introduces lattice mismatch at the interface, which inherently leads to moiré interference patterns. However, the specific shape and geometry of the resulting moiré patterns strongly depend on the mode and direction of deformation. By controlling the axial orientation and magnitude of applied strain in the way we showed, it is possible to generate novel moiré configurations. These could include 1D moiré structures, shear-induced moiré domains, and other anisotropic moiré formations.^67,68^

Different arbitrary strain profiles can be precisely designed through process-induced strain techniques. As shown in our previous works,^24,26,60^ various stressor geometries, including triangular, ring-shaped, and asymmetric profiles have been employed to control local strain distributions within 2D heterostructures. Hence, factors such as the choice of stressor material, stressor shape, and loading direction, which have been well established previously, can be easily utilized to customize moiré shapes beyond the uniform triangular subdomains. These will allow for tailored straining techniques that can reshape moiré patterns with desired symmetries.

### 3.3 Stability of Strain-Induced Moiré Domains

**Figure 4:**
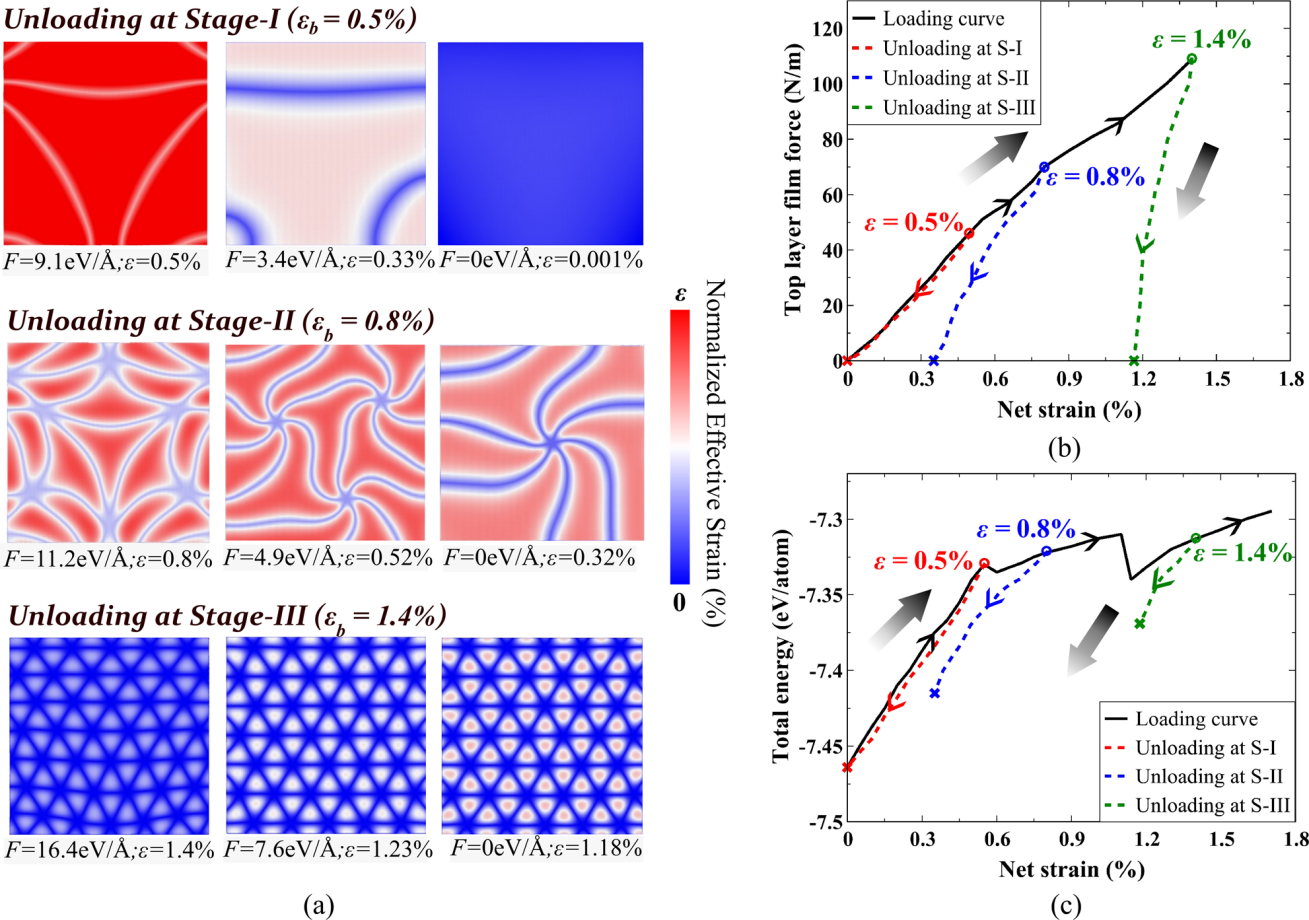
Moiré domains during unloading. (a) Effective strain contour plots illustrate the progressive unloading of the top layer for *L*_flake_ = 100 nm. Unloading simulations are conducted by gradually releasing the force from the loaded configuration. Each strain contour is adjusted using the displayed scale bar, with the upper limit representing the net residual strain present in the top layer at each unloaded state and the lower limit indicating zero strain. Unloading from stage-I (shown for ϵ_b_ = 0.5%) results in no residual strain in the fully unloaded state. The unloading trajectory from stage-II (shown for ϵ_b_ = 0.8%) depicts swirl-like spiral regions with a net residual strain upon full unloading. Releasing load from stage-III (shown for ϵ_b_ = 1.4%) exhibits no substantial change in strain distribution and the obtained triangular domains. (b) Loading–unloading curve is depicted through the film force versus net residual strain plot in the top layer. Respective black arrows pointing upward show loading and downward show unloading. The initiation of unloading from the respective stages and the completely unloaded point are, respectively, illustrated by circles and cross marks. (c) Total energy plot as a function of strain illustrates the varying energy landscapes and structural stability behavior across different stages of loading and unloading.

We examine the reversibility of this stacking order change by unloading the film and gradually removing the strain until the film force becomes zero at the edges of the film. This is done individually for all three stages, as shown in Figure 4a. The results indicate that the stability of the patterns depends on the slippage stage. In stage-I, the soliton regions gradually diminish as strain is released, eventually restoring the film to its original AB stacking configuration with no apparent residual strain. This behavior indicates that elasticity prevails in stage-I. In contrast, unloading from Stages-II and III demonstrates more complex behaviors indicative of plastic deformation as some strain remains in the film even after fully unloading the film. The skewed triangular domains in stage-II devolve into swirl-like spiral moire patterns. This type of pattern was recently observed to form in AA-stacked graphene under out-of-plane indentation loading conditions^68^ as well as the cases where a small twist angle is present between the layers.^68,69^ We show that spiral patterns are also formed in the lower energy Bernal stacking under in-plane strain and in the absence of twist. More importantly, we show that they remain stable after removing the strain. Unloading from stage-III leaves behind stable triangular moiré patterns with straight edges. Despite the complete removal of external force, the moiré patterns and associated strain contours remain intact with no configurational change in the subdomains. Our results show that spiral patterns can serve as a stable intermediate step and show later in this article that they can be used to create full triangular moire patterns.

The average film force plot as a function of strain during loading and unloading is shown in Figure 4b during loading and unloading from different stages. The curve returns to zero force during unloading from stage-I. Unloading follows the same path as loading in this stage. This is another indication of elastic behavior. However, unloading from stage-II follows a different path and results in a residual net effective strain of approximately 0.3%. In stage-III, a significant net effective strain of about 1% remains in the second layer after complete unloading from a strain of ϵ_b_ = 1.4%. The interface of graphene layers permanently locks itself into the reconfigured local subdomains if unloaded in stage-III. Analysis of the total energy of the structure further justifies the above-mentioned observations about the stability of moiré configurations when unloaded. A plot of total energy versus net effective strain is shown in Figure 4c. While no drop in energy landscape is observed in stage-I, two consequent drops are seen in transitioning to stage-II and another consequent transition from stage-II to stage-III. Unloading from stage-II does not revert the structure to the minimum energy observed at the inception of stage-II. Rather, it returns to a local minimum in stage-I, resulting in the loss of the intermediate moiré pattern obtained in stage-II and formation of a spiral pattern. Unloading from stage-III also reduces the energy to a local minimum but the extent of energy loss is noticeably less than unloading from stage-II.

### 3.4 Strain Memorization Effect

**Figure 5:**
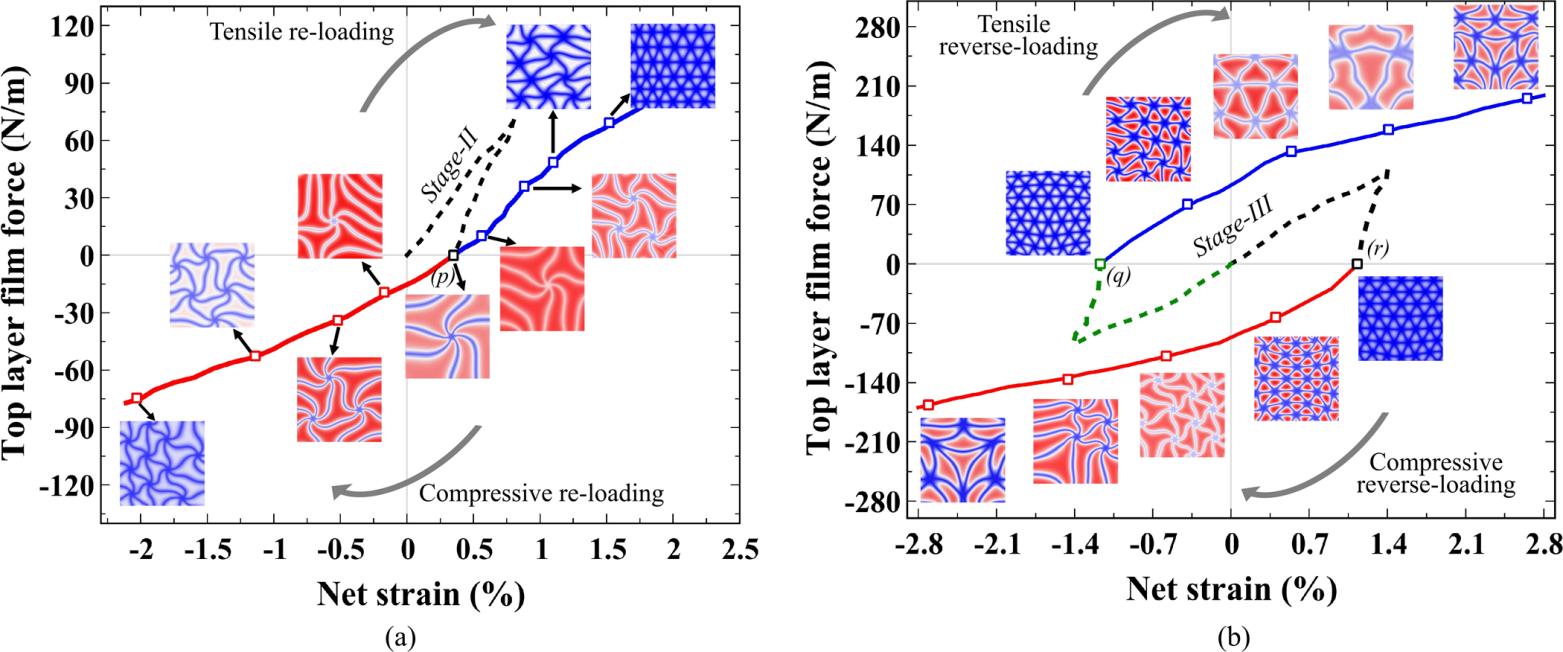
Moiré pattern evolution with strain memorization. (a) Pattern configurations plotted in film force in the top layer vs net strain during reloading of the fully unloaded configuration from stage-II. The blue and red curves represent tensile and compressive reloading, respectively, starting from the fully unloaded configuration at point *p*. Effective strain contour plots of the second layer are shown for each marked point along both loading directions. The black dotted line represents the loading–unloading curve for stage-II from Figure 4b. Negative film force indicates compressive strain. (b) Reverse loading case is shown by the force versus net strain plot for structures fully unloaded from stage-III. The black and green dotted lines indicate loading–unloading paths for stage-III under tension and compression, respectively. Fully unloaded structures are denoted by points *q* and *r*, from which reverse loading is initiated. Strain distribution contours for tensile loading to moiré patterns formed by compression, and vice versa, are shown for each denoted point along the curve. The scale bar for each plot is adjusted to the net strain present in the system.

The presented results so far are obtained with an assumption that the flake has no prior strain history. However, understanding the role of residual strains in hindering or assisting stable and consistent moiré pattern formation is important. In addition, it is advantageous to know if stable moire patterns can be obtained by reloading a film that was previously loaded. We examine the reloading behavior of fully unloaded configurations from different stages when subjected to compressive and tensile loads as summarized in Figure 5. We observe that reloading of a film that was previously loaded and unloaded from a stage-II results in forming triangular moiré domains if loading direction is preserved as shown in Figure 5a. The domains obtained by reloading are identical to those obtained by directly loading to stage-III. Contrary to reloading in the same mode, the patterns in a stage-II unloaded configuration cannot be reversed to obtain triangular moiré domains if reloaded in the opposite direction (tensile to compression reloading). As shown in Figure 5a, reloading in compression generates spiral triangular moiré patterns that resemble regular triangular shapes but have twisted edges. This indicates that there is a strain memorization effect associated with pattern formation. The strain memory allows for intermittent loading to obtain stable triangular domains if loading direction is not changed. In the event of a reversal of loading mode, strain memory results in a different kind of moiré patterns. This behavior is an indication that reapplication of strain in several steps can be used to create moire patterns or to tune their properties after fabrication.

Since stable and locked triangular moiré domains are achieved in the unloaded Stage-III, further reloading in the same direction will result in the formation of similar triangular superlattices that evolve with the additional applied strain. Thus, we further perform reverse loading on the stage-III unloaded systems (tension or compression) to investigate its effect on the developed and unchanged moire patterns. Under reverse reloading, the local atomic arrangements evolve to a moiré pattern that looks different than those obtained for structures without a strain history as shown in Figure 5b. When reverse reloading is applied (compression to tensilely formed moirés and tension to compressively formed moirés), the subdomains initially distort forming swirl-like patterns. However, with continued reverse loading the subdomains begin to form skewed domains similar to those observed during the initial loading in Figure 2. This indicates that the moiré patterns formed at stage-III exhibit significant strain memorization. Even if they lose their stable configuration due to additional strain applied, they tend to evolve into a skewed configuration while preserving their triangular construct.

### 3.5 Strain Memorization for Controlled Moiré Pattern Formation

The strain memorization effects observed during reapplication of strain from unloaded configurations represent a significant advancement in fabricating uniform moiré patterns experimentally. By utilizing the process-induced straining method discussed, strain can be applied incrementally allowing controlled formation of moiré patterns. Our results indicate that applying a smaller strain magnitude beyond the first slip (Stage-II), fully releasing the load, and then reapplying a similar strain magnitude in the same direction can yield uniform and stable moiré domains. Although our recent works show that stressor-based techniques can be designed for inducing higher strain magnitudes,^24−26,29^ it is known that applying much larger strain can be challenging due to mechanical instabilities. Our results show that a stepwise approach can be followed to eliminate the need for applying large strain magnitudes at once, making the process more manageable. However, there will be a reasonable strain limit essential to trigger such moiré domain formation, such as 0.55% strain observed for graphene. Having such smaller thresholds is well within the capability of employing strain experimentally, thus ensuring mechanical stability while fabricating strain-based moirés. Also, factors like residual strain developed during flake growth are often expected to affect strain regulation. But, residual strain in exfoliated flakes is typically minimal (0.1%) and negligible compared to the strain required for moiré formation (>0.5% for partial slip, >1% for full slip).^70^ Once partial slip occurs and moiré subdomains start forming, any minor pre-existing residual strain would become insignificant as it would be diluted by the external mechanical energy driving subdomain evolution at the interface. Since reloading occurs after surpassing these thresholds, any residual strain present is unlikely to impact moiré evolution.

The mentioned gradual strain application can be achieved using the stressor-based method, where the stressor film force is controlled by adjusting the film thickness. The loading and unloading process becomes more controllable for smaller strain magnitudes because thinner stressor films are easier to deposit and remove, as demonstrated in our previous work on trilayer graphene.^24^ Hence, the mechanisms and processes discussed in this work offer a reliable approach to achieve uniform moiré patterns, without the need for the highly uncontrollable and irreproducible method of applying interlayer twist. By adjusting the strain magnitude using this method, one can fabricate desired moiré superlattices, the mechanics of which are explained in this work. Additionally, reversing the reloading direction can result in periodic spiral moiré patterns, which are novel configurations whose features must be studied further.

Our results demonstrate that morphologically similar moiré superlattices can be obtained through interlayer heterostrain instead of twist. However, the moiré subdomains formed by strain will exhibit distinct electronic and optical properties because strain-induced characteristics differ fundamentally, as recently shown theoretically by Escudero et al. for graphene.^67^ Although their study focused on relatively larger strains (1.5–2% biaxial), the potential for fascinating properties arising from moiré patterns formed under both smaller and larger strain magnitudes merits further exploration. Moreover, the unique properties of spiral strain-induced moiré patterns as revealed in our work, could lead to novel phenomena. Importantly, the mechanisms outlined in this study can be applied to any stacked vdW material. Achieving strain-based moiré patterns in other materials such as TMDs, and investigating their properties could unlock new possibilities in the field of twistronics.

## 4 Conclusions

In summary, our study reveals that heterostraining vertically stacked graphene generates moiré patterns by introducing a lattice mismatch between the atoms of the two layers through a three-stage process, achievable under both compressive and tensile strains. We show the emergence of stable spiral patterns in stage-II, which serve as precursors to well-defined triangular subdomains in stage-III. In contrast, patterns formed in stage-I are unstable and disappear upon unloading. Additionally, our results demonstrate how nonuniform strain modalities influence the evolution of moiré patterns. A key finding of our work is the demonstration of a strain memorization effect that enables a step-by-step approach to creating and fine-tuning the morphology of moiré patterns. We also show a directional aspect of this memorization: partial moiré domains formed under tensile strain can evolve into straight-edged triangular patterns with continued tensile loading, and a similar directional dependency applies under compression. However, if the loading direction is reversed, triangular domains may form but with skewed edges that are referred to as spiral moire patterns. Attempts to remove or recreate full triangular moiré patterns through reverse reloading did not produce straight-edged patterns up to a strain magnitude of approximately 3% in the reverse direction. While additional reverse strain might evolve these patterns into triangular shapes, this possibility requires further investigation. Also, the stability of the patterns found in reversed order can be a subject of future work.

This study provides crucial insights into the interface mechanisms underlying moiré domain formation in graphene and suggests that similar behaviors may be observed in other stacked vdW materials, though at different thresholds. By identifying the precise strain conditions and thresholds that favor specific moiré configurations, this work lays the foundation for engineering material interfaces with enhanced optoelectronic properties. The ability to control and manipulate moiré domains through strain engineering has direct implications for twistronic devices, tunable excitonic platforms, and strain-controlled quantum transport in 2D heterostructures. Additionally, the strain memorization effect demonstrated here could be utilized to fabricate strain-induced moiré devices, such as reconfigurable electronic systems without requiring excessively high strain magnitudes during a single process. This could be crucial for realizing strain-engineered electronic and optical properties in a more energy-efficient and mechanically stable manner.

## Data Availability

The data supporting the findings of this study are available from the corresponding author upon reasonable request.

## Supporting Information Available

Details about the construction of deep-neural-network interatomic potential; ab initio calculations for data generation; algorithm for training, testing, and validation; additional details of the molecular statics computational method; analysis of flake size dependency for evolution of strain-induced moiré patterns; interlayer spacing contour plots; strain contour plots and interlayer slippage behavior for loading and unloading cases of additional flake sizes; and subdomain area fractions of evolving moiré patterns as a function of applied biaxial strain (PDF)

## References

[ref1] Yoo H.; Engelke R.; Carr S.; Fang S.; Zhang K.; Cazeaux P.; Sung S. H.; Hovden R.; Tsen A. W.; Taniguchi T.; Watanabe K.; Yi G.-C.; Kim M.; Luskin M.; Tadmor E. B.; Efthimios K.; Philip K. Atomic and electronic reconstruction at the van der Waals interface in twisted bilayer graphene. Nat. Mater. 2019, 18, 448–453. 10.1038/s41563-019-0346-z. 30988451

[ref2] Xiao Y.; Liu J.; Fu L. moiré is more: access to new properties of two-dimensional layered materials. Matter 2020, 3, 1142–1161. 10.1016/j.matt.2020.07.001.

[ref3] Nimbalkar A.; Kim H. Opportunities and challenges in twisted bilayer graphene: a review. Nano-Micro Lett. 2020, 12, 1–20. 10.1007/s40820-020-00464-8. PMC7770697 34138115

[ref4] Andrei E. Y.; MacDonald A. H. Graphene bilayers with a twist. Nature materials 2020, 19, 1265–1275. 10.1038/s41563-020-00840-0. 33208935

[ref5] Xie M.; MacDonald A. H. Nature of the correlated insulator states in twisted bilayer graphene. Physical review letters 2020, 124, 097601 10.1103/PhysRevLett.124.097601. 32202880

[ref6] Cao Y.; Rodan-Legrain D.; Rubies-Bigorda O.; Park J. M.; Watanabe K.; Taniguchi T.; Jarillo-Herrero P. Tunable correlated states and spin-polarized phases in twisted bilayer–bilayer graphene. Nature 2020, 583, 215–220. 10.1038/s41586-020-2260-6. 32499644

[ref7] Chen S.; He M.; Zhang Y.-H.; Hsieh V.; Fei Z.; Watanabe K.; Taniguchi T.; Cobden D. H.; Xu X.; Dean C. R.; Yankowitz M. Electrically tunable correlated and topological states in twisted monolayer–bilayer graphene. Nat. Phys. 2021, 17, 374–380. 10.1038/s41567-020-01062-6.

[ref8] Yankowitz M.; Chen S.; Polshyn H.; Zhang Y.; Watanabe K.; Taniguchi T.; Graf D.; Young A. F.; Dean C. R. Tuning superconductivity in twisted bilayer graphene. Science 2019, 363, 1059–1064. 10.1126/science.aav1910. 30679385

[ref9] Oh M.; Nuckolls K. P.; Wong D.; Lee R. L.; Liu X.; Watanabe K.; Taniguchi T.; Yazdani A. Evidence for unconventional superconductivity in twisted bilayer graphene. Nature 2021, 600, 240–245. 10.1038/s41586-021-04121-x. 34670267

[ref10] Shavit G.; Berg E.; Stern A.; Oreg Y. Theory of correlated insulators and superconductivity in twisted bilayer graphene. Physical review letters 2021, 127, 247703 10.1103/PhysRevLett.127.247703. 34951791

[ref11] Song Z.; Wang Z.; Shi W.; Li G.; Fang C.; Bernevig B. A. All magic angles in twisted bilayer graphene are topological. Physical review letters 2019, 123, 036401 10.1103/PhysRevLett.123.036401. 31386469

[ref12] Park M. J.; Kim Y.; Cho G. Y.; Lee S. Higher-order topological insulator in twisted bilayer graphene. Physical review letters 2019, 123, 216803 10.1103/PhysRevLett.123.216803. 31809156

[ref13] Fortin-Deschênes M.; Pu R.; Zhou Y.-F.; Ma C.; Cheung P.; Watanabe K.; Taniguchi T.; Zhang F.; Du X.; Xia F. Uncovering topological edge states in twisted bilayer graphene. Nano Lett. 2022, 22, 6186–6193. 10.1021/acs.nanolett.2c01481. 35900257

[ref14] Wu F.; Lovorn T.; Tutuc E.; Martin I.; MacDonald A. Topological insulators in twisted transition metal dichalcogenide homobilayers. Physical review letters 2019, 122, 086402 10.1103/PhysRevLett.122.086402. 30932597

[ref15] Devakul T.; Crépel V.; Zhang Y.; Fu L. Magic in twisted transition metal dichalcogenide bilayers. Nat. Commun. 2021, 12, 6730 10.1038/s41467-021-27042-9. 34795273 PMC8602625

[ref16] Wang L.; Shih E.-M.; Ghiotto A.; Xian L.; Rhodes D. A.; Tan C.; Claassen M.; Kennes D. M.; Bai Y.; Kim B.; Watanabe K.; Taniguchi T.; Zhu X.; Hone J.; Rubio A.; Pasupathy A. N.; Dean C. R. Correlated electronic phases in twisted bilayer transition metal dichalcogenides. Nature materials 2020, 19, 861–866. 10.1038/s41563-020-0708-6. 32572205

[ref17] Weston A.; Zou Y.; Enaldiev V.; Summerfield A.; Clark N.; Zólyomi V.; Graham A.; Yelgel C.; Magorrian S.; Zhou M.; Zultak J.; Hopkinson D.; Barinov A.; Bointon T. H.; Kretinin A.; Wilson N. R.; Beton P. H.; Fal’ko V. I.; Haigh S. J.; Gorbachev R. Atomic reconstruction in twisted bilayers of transition metal dichalcogenides. Nature Nanotechnol. 2020, 15, 592–597. 10.1038/s41565-020-0682-9. 32451502

[ref18] Tran K.; Choi J.; Singh A.; et al. Moire and beyond in transition metal dichalcogenide twisted bilayers. 2D Materials 2021, 8, 022002 10.1088/2053-1583/abd3e7.

[ref19] Lau C. N.; Bockrath M. W.; Mak K. F.; Zhang F. Reproducibility in the fabrication and physics of moiré materials. Nature 2022, 602, 41–50. 10.1038/s41586-021-04173-z. 35110759

[ref20] Cai L.; Yu G. Fabrication strategies of twisted bilayer graphenes and their unique properties. Adv. Mater. 2021, 33, 2004974 10.1002/adma.202004974. 33615593

[ref21] Liu C.; Li Z.; Qiao R.; Wang Q.; Zhang Z.; Liu F.; Zhou Z.; Shang N.; Fang H.; Wang M.; Liu Z.; Feng Z.; Cheng Y.; Wu H.; Gong D.; Yu D.; Wang E.; Wang Z.-J.; Liu K. Designed growth of large bilayer graphene with arbitrary twist angles. Nat. Mater. 2022, 21, 1263–1268. 10.1038/s41563-022-01361-8. 36109673

[ref22] Chu C.-M.; Woon W.-Y. Growth of twisted bilayer graphene through two-stage chemical vapor deposition. Nanotechnology 2020, 31, 435603 10.1088/1361-6528/aba39e. 32634795

[ref23] Ma W.; Chen M.-L.; Yin L.; Liu Z.; Li H.; Xu C.; Xin X.; Sun D.-M.; Cheng H.-M.; Ren W. Interlayer epitaxy of wafer-scale high-quality uniform AB-stacked bilayer graphene films on liquid Pt3Si/solid Pt. Nat. Commun. 2019, 10, 2809 10.1038/s41467-019-10691-2. 31243279 PMC6594936

[ref24] Dey A.; Azizimanesh A.; Wu S. M.; Askari H. Uniaxial Strain-Induced Stacking Order Change in Trilayer Graphene. ACS Appl. Mater. Interfaces 2024, 16, 8169–8183. 10.1021/acsami.3c19101. 38295436 PMC10875650

[ref25] Hou Y.; Zhou J.; Xue M.; Yu M.; Han Y.; Zhang Z.; Lu Y. Strain Engineering of Twisted Bilayer Graphene: The Rise of Strain-Twistronics. Small 2024, 2311185 10.1002/smll.202311185. PMC12272032 38616775

[ref26] Azizimanesh A.; Dey A.; Chowdhury S. A.; Wenner E.; Hou W.; Peña T.; Askari H.; Wu S. M. Strain engineering in 2D hBN and graphene with evaporated thin film stressors. Appl. Phys. Lett. 2023, 123, 043504 10.1063/5.0153935.

[ref27] Peña T.; Dey A.; Chowdhury S. A.; Azizimanesh A.; Hou W.; Sewaket A.; Watson C.; Askari H.; Wu S. M. moiré engineering in 2D heterostructures with process-induced strain. Appl. Phys. Lett. 2023, 122, 143101 10.1063/5.0142406.

[ref28] Thompson S. E.; Armstrong M.; Auth C.; Alavi M.; Buehler M.; Chau R.; Cea S.; Ghani T.; Glass G.; Hoffman T. A 90-nm logic technology featuring strained-silicon. IEEE Trans. Electron Devices 2004, 51, 1790–1797. 10.1109/TED.2004.836648.

[ref29] Peña T.; Chowdhury S. A.; Azizimanesh A.; Sewaket A.; Askari H.; Wu S. M. Strain engineering 2D MoS2 with thin film stress capping layers. 2D Materials 2021, 8, 045001 10.1088/2053-1583/ac08f2.

[ref30] Chowdhury S. A.; Inzani K.; Peña T.; Dey A.; Wu S. M.; Griffin S. M.; Askari H. Mechanical properties and strain transfer behavior of molybdenum ditelluride (MoTe2) thin films. J. Eng. Mater. Technol. 2022, 144, 011006 10.1115/1.4051306.

[ref31] Hou W.; Azizimanesh A.; Dey A.; Yang Y.; Wang W.; Shao C.; Wu H.; Askari H.; Singh S.; Wu S. M. Strain engineering of vertical molybdenum ditelluride phase-change memristors. Nat. Electron. 2023, 7, 8–16. 10.1038/s41928-023-01071-2.

[ref32] Wen T.; Zhang L.; Wang H.; Weinan E.; Srolovitz D. J. Deep potentials for materials science. Mater. Futures 2022, 1, 022601 10.1088/2752-5724/ac681d.

[ref33] Unke O. T.; Chmiela S.; Sauceda H. E.; Gastegger M.; Poltavsky I.; Schütt K. T.; Tkatchenko A.; Müller K.-R. Machine Learning Force Fields. Chem. Rev. 2021, 121, 10142–10186. 10.1021/acs.chemrev.0c01111. 33705118 PMC8391964

[ref34] Behler J. Perspective: Machine learning potentials for atomistic simulations. J. Chem. Phys. 2016, 145, 170901 10.1063/1.4966192. 27825224

[ref35] Deringer V. L.; Caro M. A.; Csányi G. Machine learning interatomic potentials as emerging tools for materials science. Adv. Mater. 2019, 31, 1902765 10.1002/adma.201902765. 31486179

[ref36] Leconte N.; Javvaji S.; An J.; Samudrala A.; Jung J. Relaxation effects in twisted bilayer graphene: A multiscale approach. Phys. Rev. B 2022, 106, 115410 10.1103/PhysRevB.106.115410.

[ref37] Liu X.; Peng R.; Sun Z.; Liu J. moiré phonons in magic-angle twisted bilayer graphene. Nano Lett. 2022, 22, 7791–7797. 10.1021/acs.nanolett.2c02010. 36170965 PMC9562463

[ref38] Que Z.-X.; Li S.-Z.; Huang B.; Yang Z.-X.; Zhang W.-B. Ultra-flat bands at large twist angles in group-V twisted bilayer materials. J. Chem. Phys. 2024, 160, 194710 10.1063/5.0197757. 38767261

[ref39] Wang H.; Zhang L.; Han J.; Weinan E. DeePMD-kit: A deep learning package for many-body potential energy representation and molecular dynamics. Comput. Phys. Commun. 2018, 228, 178–184. 10.1016/j.cpc.2018.03.016.

[ref40] Zhang Y.; Wang H.; Chen W.; Zeng J.; Zhang L.; Wang H.; Weinan E. DP-GEN: A concurrent learning platform for the generation of reliable deep learning based potential energy models. Comput. Phys. Commun. 2020, 253, 107206 10.1016/j.cpc.2020.107206.

[ref41] Giannozzi P.; Baroni S.; Bonini N.; Calandra M.; Car R.; Cavazzoni C.; Ceresoli D.; Chiarotti G. L.; Cococcioni M.; Dabo I. QUANTUM ESPRESSO: a modular and open-source software project for quantum simulations of materials. J. Phys.: Condens. Matter 2009, 21, 395502 10.1088/0953-8984/21/39/395502. 21832390

[ref42] Dey A.; Baraiya B. A.; Adhikary S.; Jha P. K. First-principles calculations of the effects of edge functionalization and size on the band gap of be3n2 nanoribbons: Implications for nanoelectronic devices. ACS Applied Nano Materials 2021, 4, 493–502. 10.1021/acsanm.0c02809.

[ref43] Dey A.; Sharma R.; Dar S. A. An extensive investigation of structural, electronic, thermoelectric and optical properties of bi-based half-Huesler alloys by first principles calculations. Materials Today Communications 2020, 25, 101647 10.1016/j.mtcomm.2020.101647.

[ref44] Kumar V.; Dey A.; Thomas S.; Zaeem M. A.; Roy D. R. Hydrogen-induced tunable electronic and optical properties of a two-dimensional penta-Pt 2 N 4 monolayer. Phys. Chem. Chem. Phys. 2021, 23, 10409–10417. 10.1039/D1CP00681A. 33889892

[ref45] Mostafa A.; Vu L.; Guo Z.; Shargh A. K.; Dey A.; Askari H.; Abdolrahim N. Phase-transformation assisted twinning in Molybdenum nanowires. Comput. Mater. Sci. 2024, 244, 113273 10.1016/j.commatsci.2024.113273.

[ref46] Hou W.; Chowdhury S. A.; Dey A.; Watson C.; Peña T.; Azizimanesh A.; Askari H.; Wu S. M. Nonvolatile Ferroelastic Strain from Flexoelectric Internal Bias Engineering. Physical Review Applied 2022, 17, 024013 10.1103/PhysRevApplied.17.024013.

[ref47] Thompson A. P.; Aktulga H. M.; Berger R.; Bolintineanu D. S.; Brown W. M.; Crozier P. S.; in’t Veld P. J.; Kohlmeyer A.; Moore S. G.; Nguyen T. D.; Shan R.; Stevens M. J.; Tranchida J.; Trott C.; Plimpton S. J. LAMMPS-a flexible simulation tool for particle-based materials modeling at the atomic, meso, and continuum scales. Comput. Phys. Commun. 2022, 271, 108171 10.1016/j.cpc.2021.108171.

[ref48] Kumar P.; Dey A.; Roques J.; Assaud L.; Franger S.; Parida P.; Biju V. Photoexfoliation synthesis of 2D materials. ACS Materials Letters 2022, 4, 263–270. 10.1021/acsmaterialslett.1c00651.

[ref49] Surana M.; Ahmed T.; Admal N. C. Interface mechanics of 2D materials on metal substrates. Journal of the Mechanics and Physics of Solids 2022, 163, 104831 10.1016/j.jmps.2022.104831.

[ref50] Sun Z.; Raji A.-R. O.; Zhu Y.; Xiang C.; Yan Z.; Kittrell C.; Samuel E.; Tour J. M. Large-area Bernal-stacked bi-, tri-, and tetralayer graphene. ACS Nano 2012, 6, 9790–9796. 10.1021/nn303328e. 23110694

[ref51] Lu C.-C.; Lin Y.-C.; Liu Z.; Yeh C.-H.; Suenaga K.; Chiu P.-W. Twisting bilayer graphene superlattices. ACS Nano 2013, 7, 2587–2594. 10.1021/nn3059828. 23448165

[ref52] Nakatsuji N.; Kawakami T.; Koshino M. Multiscale lattice relaxation in general twisted trilayer graphenes. Physical Review X 2023, 13, 041007 10.1103/PhysRevX.13.041007.

[ref53] Dey A.; Chowdhury S. A.; Peña T.; Singh S.; Wu S. M.; Askari H. An Atomistic Insight into moiré Reconstruction in Twisted Bilayer Graphene beyond the Magic Angle. ACS Applied Engineering Materials 2023, 1, 970–982. 10.1021/acsaenm.2c00259. 37008886 PMC10043875

[ref54] Alden J. S.; Tsen A. W.; Huang P. Y.; Hovden R.; Brown L.; Park J.; Muller D. A.; McEuen P. L. Strain solitons and topological defects in bilayer graphene. Proc. Natl. Acad. Sci. U. S. A. 2013, 110, 11256–11260. 10.1073/pnas.1309394110. 23798395 PMC3710814

[ref55] Kumar H.; Dong L.; Shenoy V. B. Limits of coherency and strain transfer in flexible 2D van der Waals heterostructures: formation of strain solitons and interlayer debonding. Sci. Rep. 2016, 6, 21516 10.1038/srep21516. 26867496 PMC4751462

[ref56] Bai H.; Bao H.; Li Y.; Xu H.; Li S.; Ma F. One-Dimensional Strain Solitons Manipulated Superlubricity on Graphene Interface. J. Phys. Chem. Lett. 2022, 13, 7261–7268. 10.1021/acs.jpclett.2c02066. 35914178

[ref57] Wang K.; Qu C.; Wang J.; Ouyang W.; Ma M.; Zheng Q. Strain engineering modulates graphene interlayer friction by moiré pattern evolution. ACS Appl. Mater. Interfaces 2019, 11, 36169–36176. 10.1021/acsami.9b09259. 31486630

[ref58] Hang Y.; Zhang Z. Heterostrain-induced flat bands in untwisted bilayer graphene. Acta Mech. Sin. 2024, 40, 123176 10.1007/s10409-023-23176-x.

[ref59] Dong J. T.; Inbar H. S.; Dempsey C. P.; Engel A. N.; Palmstro̷m C. J. Strain Solitons in an Epitaxially Strained van der Waals-like Material. Nano Lett. 2024, 24, 4493–4497. 10.1021/acs.nanolett.4c00382. 38498733 PMC11036392

[ref60] Zhang Y.; Hossain M. A.; Hwang K. J.; Ferrari P. F.; Maduzia J.; Peña T.; Wu S. M.; Ertekin E.; van der Zande A. M. Patternable Process-Induced Strain in 2D Monolayers and Heterobilayers. ACS Nano 2024, 18, 4205–4215. 10.1021/acsnano.3c09354. 38266246

[ref61] Kazmierczak N. P.; Van Winkle M.; Ophus C.; Bustillo K. C.; Carr S.; Brown H. G.; Ciston J.; Taniguchi T.; Watanabe K.; Bediako D. K. Strain fields in twisted bilayer graphene. Nature materials 2021, 20, 956–963. 10.1038/s41563-021-00973-w. 33859383

[ref62] Gadelha A. C.; Ohlberg D. A.; Rabelo C.; Neto E. G.; Vasconcelos T. L.; Campos J. L.; Lemos J. S.; Ornelas V.; Miranda D.; Nadas R.; Santana F. C.; Watanabe K.; Taniguchi T.; Campos L. C.; Cançado L. G.; Medeiros-Ribeiro G.; Jorio A. Localization of lattice dynamics in low-angle twisted bilayer graphene. Nature 2021, 590, 405–409. 10.1038/s41586-021-03252-5. 33597759

[ref63] Barbosa T. C.; Gadelha A. C.; Ohlberg D. A.; Watanabe K.; Taniguchi T.; Medeiros-Ribeiro G.; Jorio A.; Campos L. C. Raman spectra of twisted bilayer graphene close to the magic angle. 2D Materials 2022, 9, 025007 10.1088/2053-1583/ac4af9.

[ref64] Xu Y.; Li X.; Dong J. Infrared and Raman spectra of AA-stacking bilayer graphene. Nanotechnology 2010, 21, 065711 10.1088/0957-4484/21/6/065711. 20061600

[ref65] Poncharal P.; Ayari A.; Michel T.; Sauvajol J.-L. Raman spectra of misoriented bilayer graphene. Phys. Res. B Condens. Matter Mater. 2008, 78, 113407 10.1103/PhysRevB.78.113407.

[ref66] Jorio A.; Cançado L. G. Raman spectroscopy of twisted bilayer graphene. Solid State Commun. 2013, 175, 3–12. 10.1016/j.ssc.2013.08.008.

[ref67] Escudero F.; Sinner A.; Zhan Z.; Pantaleón P. A.; Guinea F. Designing moiré patterns by strain. Physical Review Research 2024, 6, 023203 10.1103/PhysRevResearch.6.023203.

[ref68] Zhu G.; Liu R.; Tang C.; Wang L. Dynamic tuning of moiré superlattice morphology by out-of-plane deformation. Appl. Phys. Lett. 2024, 124, 173508 10.1063/5.0202712.

[ref69] Mesple F.; Walet N. R.; Trambly de Laissardière G.; Guinea F.; Došenović D.; Okuno H.; Paillet C.; Michon A.; Chapelier C.; Renard V. T. Giant atomic swirl in graphene bilayers with biaxial heterostrain. Adv. Mater. 2023, 35, 2306312 10.1002/adma.202306312. 37615204

[ref70] Michail A.; Delikoukos N.; Parthenios J.; Galiotis C.; Papagelis K. Optical detection of strain and doping inhomogeneities in single layer MoS2. Appl. Phys. Lett. 2016, 108, 173102 10.1063/1.4948357.

