# Supplementary material for: Memorization of Strain-Induced Moiré Patterns in Vertical van der Waals Materials
Source: ACS Appl Mater Interfaces. 2025 Mar 4;17(10):16223–33. doi: 10.1021/acsami.4c22462 (PMC11912196; doi:10.1021/acsami.4c22462)
Supplement: Supplementary file 1 — am4c22462_si_001.pdf [file am4c22462_si_001.pdf]

Supporting Information

Memorization of strain-induced moiré patterns in vertical van der Waals material

Aditya Dey*,[1,a)] Nazmul Hasan,[2] Stephen M. Wu,[2,b)] and Hesam Askari[1]

[1)] *Department of Mechanical Engineering, University of Rochester, Rochester, New York 14611, United States*

[2)] *Department of Electrical and Computer Engineering, University of Rochester, Rochester, New York 14620, United States*

[a)] Electronic mail: adey2@ur.rochester.edu

[b)] Department of Physics and Astronomy, University of Rochester, Rochester, New York 14611, United States

I. CONSTRUCTION OF A DEEP-NEURAL-NETWORK INTERATOMIC POTENTIAL

A. Ab initio calculations for data generation

For the training and evaluation of the deep neural network potential, we prepared separate datasets for training and testing, ensuring a diverse and comprehensive set of atomic configurations. These datasets were generated using density-functional theory (DFT) calculations, specifically leveraging the Quantum Espresso (QE) open-source package [1]. We utilized the Perdew-Burke-Ernzerhof (PBE) form within the generalized gradient approximation (GGA) framework as the exchange-correlation functional [2–4], which provides a balance between accuracy and computational efficiency for a wide range of material systems [5, 6]. Ultrasoft pseudopotentials were employed to accurately describe the ion-electron interactions for carbon atoms, which are critical for capturing the subtleties of bond formation and breaking. To incorporate van der Waals (vdW) interactions, essential for modeling layered materials like graphene, we applied the semi-empirical Grimme functional [7]. The plane-wave energy cutoff was meticulously set at 55 Ry, while the charge density cutoff was defined at 450 Ry, ensuring that the calculations captured the fine details of electron distribution. For AIMD (ab initio molecular dynamics) simulations, an energy convergence threshold of 10^{-7} eV was enforced to guarantee the precision of the dynamic simulations. Before conducting AIMD simulations, we performed a thorough relaxation of the initial configurations for each structure until the atomic forces were reduced to less than 0.01 eV/Å, ensuring the systems were in a near-equilibrium state. To accurately sample the electronic properties, the Brillouin zone was sampled using Monkhorst-Pack k-point grids [8], set at $8 \times 8 \times 1$ for bilayer graphene (BLG) structures and $4 \times 4 \times 1$ for twisted BLG structures, optimizing the balance between computational cost and accuracy.

AIMD calculations were conducted at a range of finite temperatures (10K, 300K, and 500K) across all structures, including both strained and unstrained AB bilayer graphene (BLG) and twisted bilayer graphene structures with twist angles spanning from 21.79° to 6.1°. These temperatures were chosen to capture the behavior of graphene under various thermal conditions, from near-zero temperature stability to room temperature dynamics and

higher temperature effects that may induce phase transitions. For strained configurations of BLG, we applied controlled small strains (ranging from 0.5% to 1.5%) to the crystal lattice, allowing the internal atomic coordinates to fully relax and achieve an optimized strained configuration, accurately reflecting realistic experimental conditions. These strained structures were strategically included in the training set to enhance the ability of the model to comprehend atomic interactions under stress, thus improving its predictive power for strained systems. Additionally, we incorporated moiré superlattice structures into the training dataset to ensure the model could simulate and predict the formation and evolution of strain-induced moiré patterns, a critical aspect for understanding the electronic and mechanical properties of twisted bilayer graphene. To further enrich the dataset, several perturbed structures were generated using AIMD at different temperatures, applying small atomic displacements to these initially relaxed configurations. Each AIMD simulation was run for 100 fs per structure, capturing a sufficient number of atomic configurations to build a robust and comprehensive dataset for training, ensuring reliability of the model in predicting complex atomic behaviors.

B. Training, testing, and validation

In this work, we utilize the DeePMD-kit package for developing the deep neural network potential [9, 10]. The total potential energy E of the system is calculated as the aggregate of individual atomic energies:

$$E = \sum E_i \tag{1}$$

The energy of each atom E_i is defined by its local atomic environment, expressed as:

$$E_i = E_s(i)\left(R_i, \{R_j | j \in N_{R_c}(i)\}\right) \tag{2}$$

Here, N_{R_c} indicates the index of atoms within the cutoff radius R_c, and s_i specifies the chemical species of the atom i. Atomic coordinates contribute to the feature matrix D_α^{ij} as follows:

$$D_\alpha^{ij} = \begin{cases} \left\{ \frac{1}{R_{ij}}, \frac{x_{ij}}{R_{ij}}, \frac{y_{ij}}{R_{ij}}, \frac{z_{ij}}{R_{ij}} \right\}, & \text{if } R_{ij} \leq R_{cs}; \\ \left\{ \frac{1}{R_{ij}} \right\}, & \text{if } R_{cs} \leq R_{ij} \leq R_c. \end{cases} \tag{3}$$

To train the model, we configure the embedding and fitting neural networks with layer sizes set to $\{25, 50, 100\}$ and $\{240, 240, 240\}$, respectively. This setup includes adjusting for long-range Coulomb and van der Waals forces by applying a substantial cutoff radius of 10 Å. We also define several prefactors for the loss functions—$p_e^\star$, p_{limit}^e, $p_f^\star$, p_{limit}^f, $p_v^\star$, and p_{limit}^v—with values set to 0.02, 8, 1000, 2, 0.8, and 1, respectively. A training duration of two million steps ensures the model reaches convergence.

After compiling the necessary dataset, we proceeded with rigorous testing and validation steps to ensure that the machine-learned potential accurately minimizes the loss function by comparing the predicted values against the actual data. This process involved extensive evaluation to verify the ability of the model to predict atomic interactions with high fidelity. To assess the accuracy of our machine-learned interatomic potential (MLIP), we conducted a detailed comparison between the total energies and atomic forces derived from density functional theory (DFT) calculations and those predicted by the MLIP. This comparison is essential for determining the precision of the model in replicating the complex interactions within the material systems. The results demonstrated a strong correlation between the DFT and MLIP predictions, as clearly illustrated in the parity plots (Fig. S1). These plots show a close alignment between the predicted and actual values, underscoring the robust predictive capability of our model. Such strong agreement is a testament to the effectiveness of the model in capturing the intricacies of atomic-scale interactions. This validation phase is critical not only for fine-tuning the model parameters but also for enhancing its overall reliability, making it a powerful tool for materials design and the simulation of complex atomic interactions. The successful validation builds confidence in the ability of MLIP to accurately simulate and predict the behavior of advanced material systems under various conditions, paving the way for its application in more sophisticated and predictive materials science research.

II. MOLECULAR STATICS (MS) CALCULATIONS

The MS method was employed at a temperature of T = 0 K, where we applied a constant incremental biaxial strain in both the x and y directions to the top layer [11–13]. This biaxial strain, defined as $\epsilon_b = \sqrt{\epsilon_x^2 + \epsilon_y^2}$, was gradually increased up to a final magnitude of

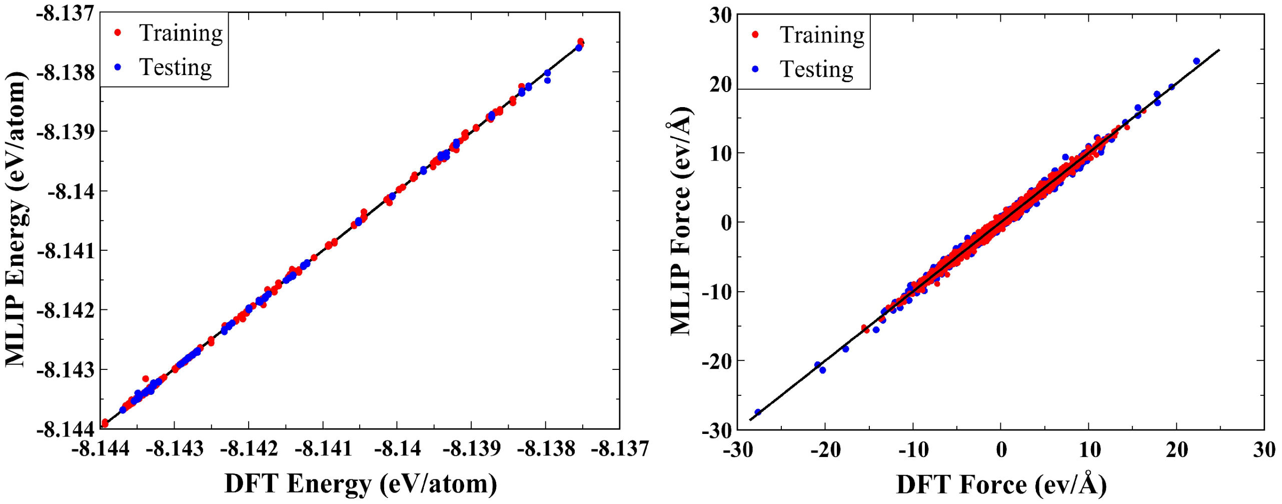

(a)

(b)

FIG. S1: Validation of the deep neural network based MLIP potential. Parity plots of MLIP predicted values and DFT calculated values for (a) total energy and (b) atomic forces of training and testing datasets.

2.1%. Following each strain increment, the atoms in the top layer were held stationary at the prescribed strain level, and energy minimization was conducted using the conjugate gradient method for each increment. Finally, the Ovito open visualization tool was utilized to analyze the simulation results [14]. The atomic strain feature in Ovito allowed us to compute the average strain on the bottom layer and visualize the local strain distribution effectively.

We perform molecular statics simulations using the open-source LAMMPS software, enhanced by integrating the DeepMD toolkit within LAMMPS binary for employing the DeepMD ML interatomic potential [15]. Our simulations focused on modeling the initial DFT-relaxed AB bilayer graphene structure as square planar flakes, with dimensions ranging from 25 nm to 250 nm in length. We implemented free boundary conditions to accommodate the inequivalent strain applied exclusively to the top graphene layer. The molecular statics method was employed at a temperature of T = 0 K, where we applied a constant incremental biaxial strain in both the x and y directions to the top layer. The biaxial strain, defined as $\epsilon_b = \sqrt{\epsilon_x^2 + \epsilon_y^2}$, was gradually increased up to a final strain magnitude, which varied depending on the loading conditions. After each incremental strain application, the atoms in

the top layer were held stationary at the specified strain level, and an energy minimization process was performed using the conjugate gradient method [16]. This method was chosen for its efficiency in reaching the minimum energy configuration, ensuring that the system was stable at each strain increment.

To interpret the simulation results, we utilized the Ovito open visualization tool, which provided powerful features for analyzing atomic structures. Specifically, the atomic strain feature in Ovito enabled us to compute the average strain on the bottom graphene layer, offering insights into how strain was transferred from the top to the bottom layer. Additionally, Ovito allowed us to visualize the local strain distribution across the graphene flake, highlighting regions of concentrated strain and potential sites for local structural changes. This detailed analysis was crucial for understanding the mechanical behavior at the interface under biaxial strain that facilitated the formation of moiré sub-domains.

III. FLAKE SIZE DEPENDENCY FOR EVOLUTION OF STRAIN-INDUCED MOIRÉS

It is important to emphasize that the formation of moiré patterns is not merely an edge effect associated with the confined dimensions of the graphene flakes. This is demonstrated by the consistent evolution of moiré patterns across different flake lengths. A comparison of moiré superlattice lengths, both underdeveloped and fully developed, in flakes of 100 nm and 250 nm through all loading stages reveals that the evolution of moiré patterns and the mechanism of triangular domain formation are independent of flake size. While strain localizations and solitons often originate at the edges due to unique boundary conditions—where atoms are less constrained and more responsive to external stresses—these phenomena are not limited to edge effects alone. The responsiveness of edge atoms allows them to more readily accommodate and dissipate strain through rearrangements, facilitating the early formation of strain-relieving features such as solitons and relaxed domains as the material adjusts to minimize energy under external load. This structural reorganization is crucial for the material to accommodate external stresses without catastrophic failure, enabling it to adapt through the formation of incommensurate domains.

However, the overall number of fully developed moiré patterns and the attainment of

the desired superlattice lengths are influenced by the dimensions of the flakes. This size dependence is analyzed through a detailed evaluation of interlayer shear resistance, which varies with flake geometry. For example, flake sizes smaller than the desired moiré pattern length can impede the full development of moiré structures. We present a comparative plot showing how the ratio of flake length to moiré length ($L_{flake}/L_{moiré}$) influences the expected moiré superlattice length ($L_{expected}/L_{actual}$). Our analysis reveals that flake sizes approximately eight times the desired moiré pattern length show optimal alignment with the expected superlattice dimensions. The discrepancies in $L_{moiré}$ for smaller $L_{flake}/L_{moiré}$ ratios are attributed to incomplete slippage phenomena, leading to residual strain transfer to the bottom layer. This analysis underscores the pivotal role of flake size in governing interface dynamics and moiré pattern formation, offering valuable insights into the underlying mechanisms that drive these complex phenomena. Understanding this relationship is critical for optimizing material design and engineering applications that rely on precise control of moiré patterns in van der Waals materials.

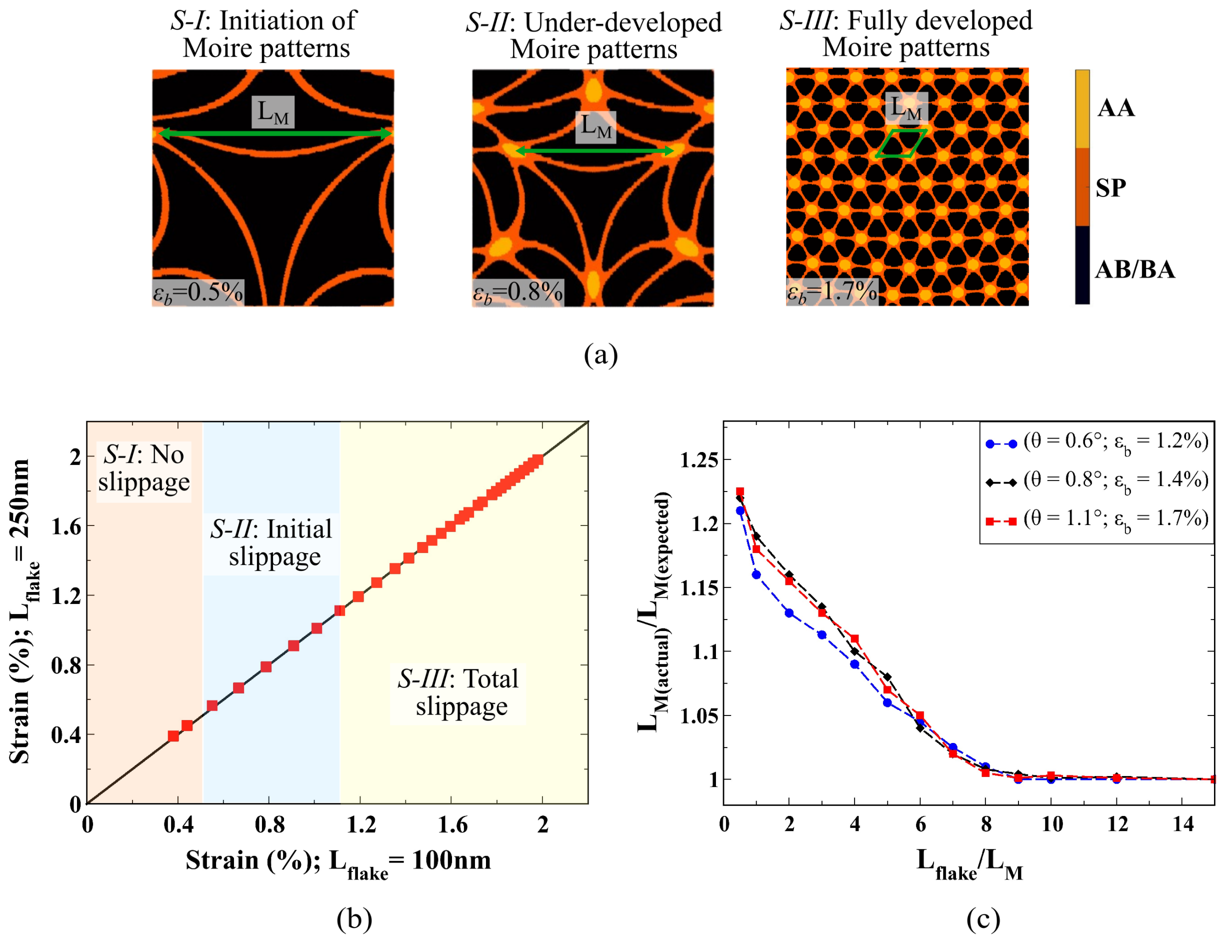

FIG. S2: (a) Estimation of moiré superlattice lengths (L_M) for emerging and fully evolved moiré patterns across different stages (L_{flake} = 100 nm). (b) Comparative analysis showing the alignment of L_M between 100 nm and 250 nm flakes across three loading stages, indicating that strain-induced moiré formation is independent of flake size. (c) Plot of the flake length-to-moiré length ratio ($L_{flake}/L_{moiré}$) versus the expected superlattice length ($L_{expected}/L_{actual}$). Flake sizes larger than eight times the desired moiré pattern length exhibit optimal alignment, while smaller flakes show discrepancies due to incomplete interlayer slippage and residual strain transfer

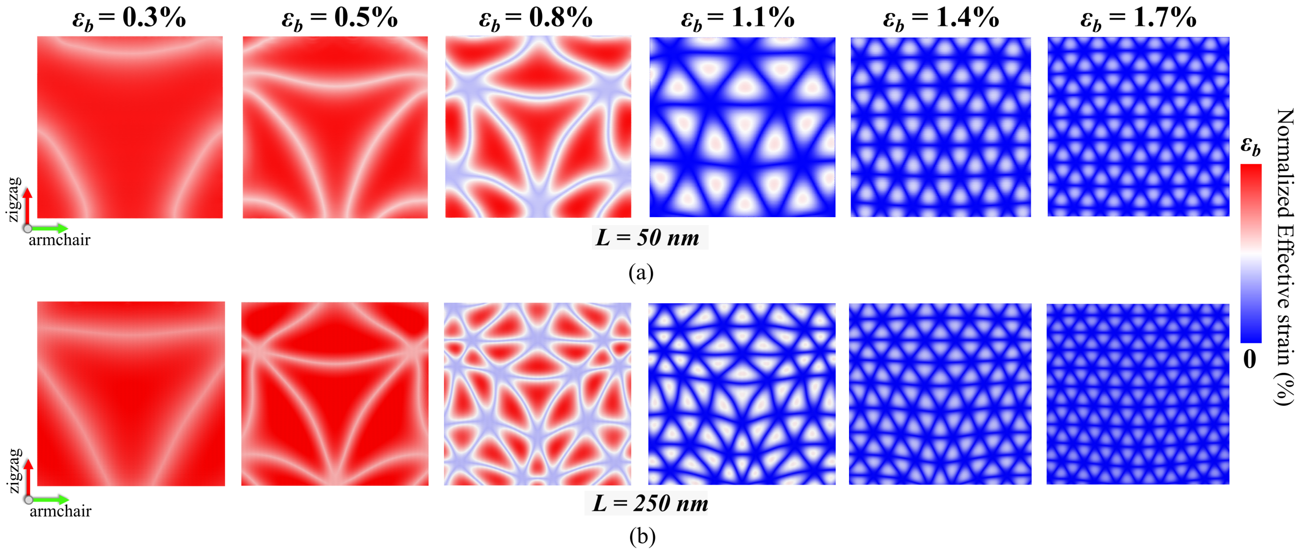

FIG. S3: Effective strain distribution contour plots of the bottom layer for (a) 50nm and (b) 250nm flakes while applying biaxial tension. Each contour is calibrated using the displayed scale bar, with the upper limit representing the biaxial strain applied to the top layer (ϵ_b) and the lower limit indicating zero strain.

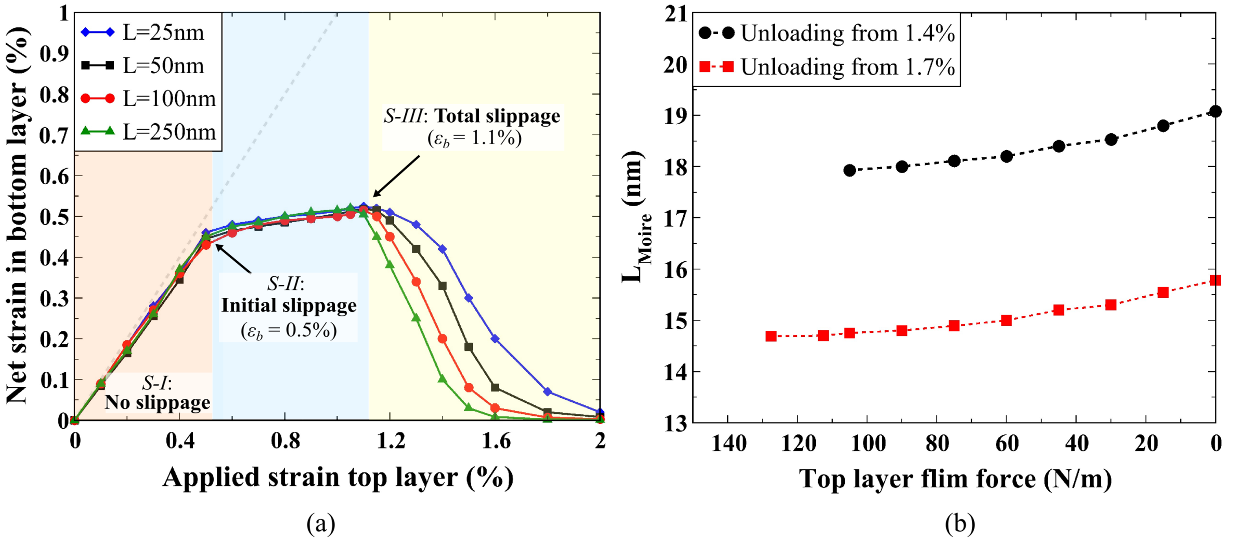

FIG. S4: (a)Net strain in the bottom layer as a function of the applied strain in the top layer shown for different flake lengths (25nm, 50nm, 100 nm, and 250 nm). The three-stage loading behavior, i.e.: elastic deformation (stage I (S-I)), initial slippage (stage II (S-II)), and complete slippage/failure (stage III (S-III)) is consistent across different flake lengths. (b) Comparison of change in moiré superlattice lengths while unloading from 1.4% and 1.7% strain. The negligible change in length till the completely unloaded state shows the stability of the formed moiré domains at this loading stage.

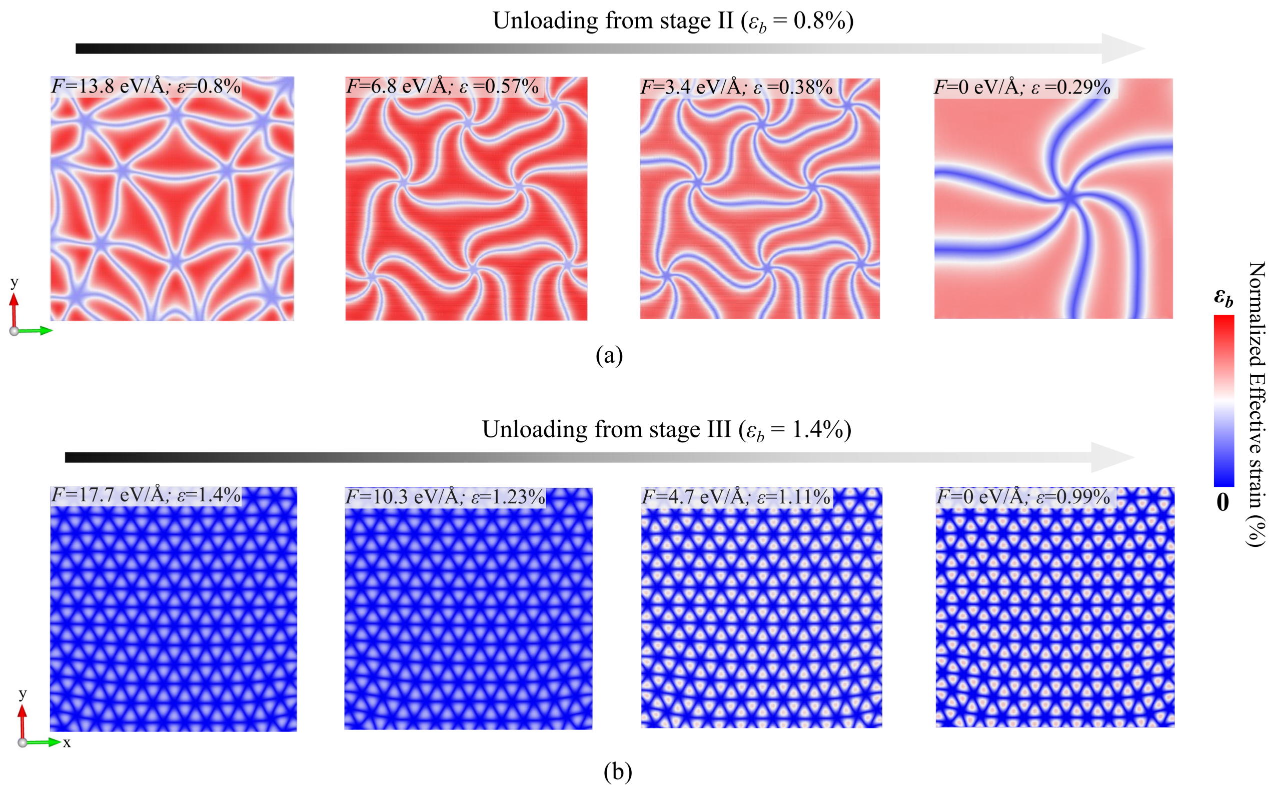

FIG. S5: Von-Mises strain contour plots while unloading the 250nm flake from (a) 0.8% in Stage-II and (b) 1.4% in Stage III. Each strain contour is adjusted using the displayed scale bar, with the upper limit representing the net residual strain present in the top layer at each unloaded configuration and the lower limit indicating zero strain. The sub-domain formations (including the swirl-like patterns) while unloading follow a similar behavior to 100nm flake the swirl-like domains, showing that the mechanism is independent of flake length.

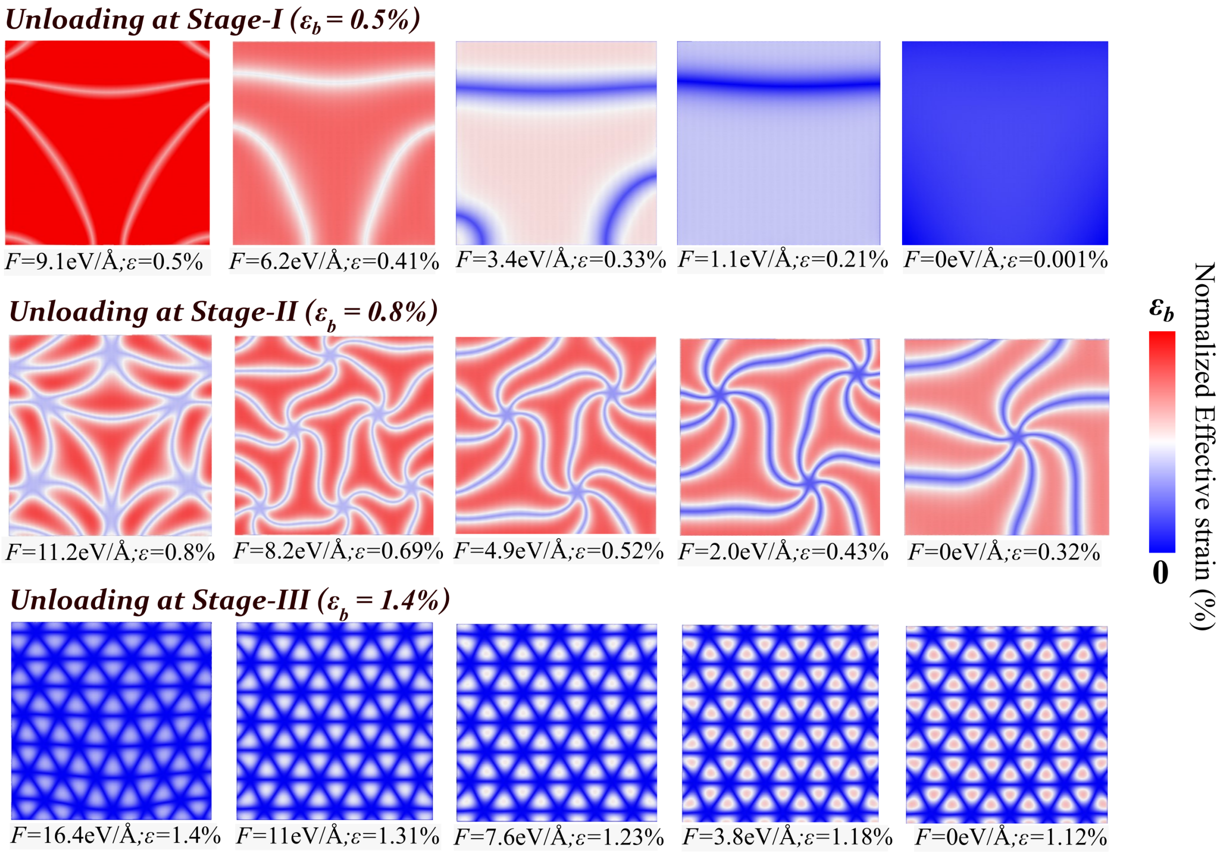

FIG. S6: Effective strain contour plots show the progressive unloading of the top layer for L_{flake} = 100 nm. The scale bar indicates residual strain, with the upper limit showing the net residual strain and the lower limit indicating zero strain. (a) Unloading from stage I (ϵ_b = 0.5%) shows gradual distortion, leading to no residual strain. (b) Unloading from stage II (ϵ_b = 0.8%) reveals spiral regions with residual strain after full unloading. (c) Unloading from stage III (ϵ_b = 1.4%) shows stable moiré patterns with no significant change in localized strain distribution.

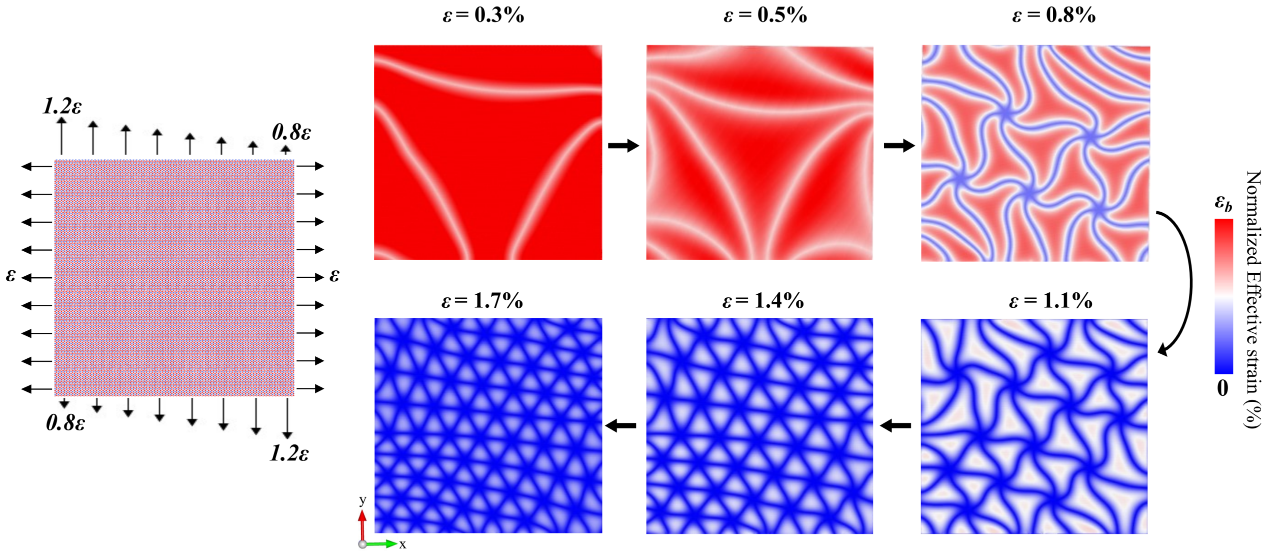

(a)

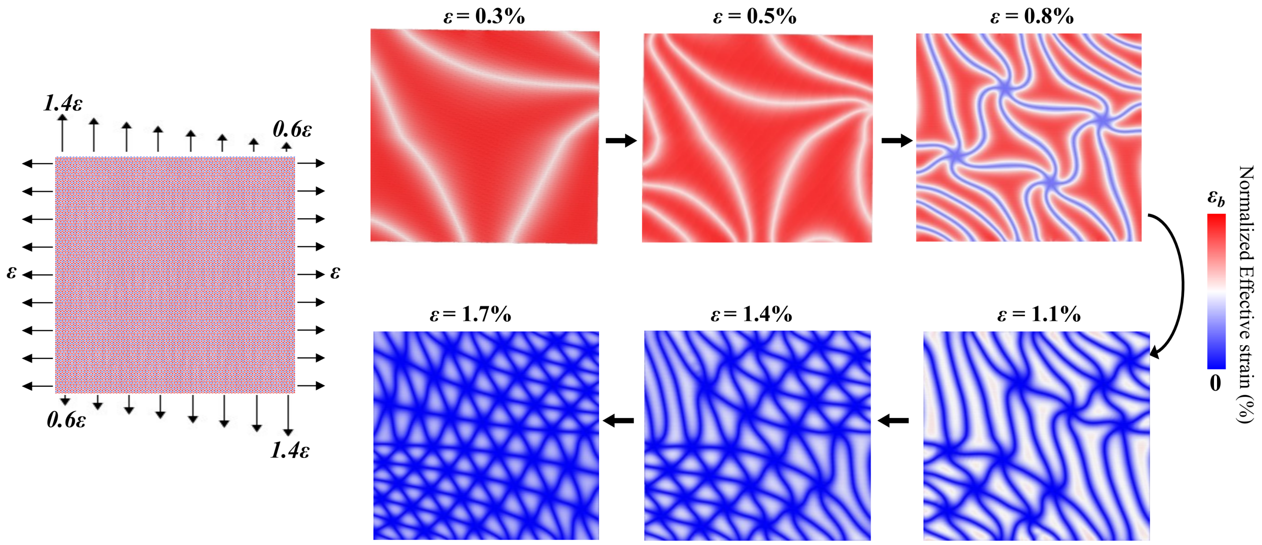

(b)

FIG. S7: Effective strain contour plots for non-uniform loading conditions: (a) Case II: Uniform along x-axis and distributed load (0.8ϵ-1.2ϵ) along y-axis and (b) Case III: Uniform along x-axis and distributed load (0.6ϵ-1.4ϵ) along y-axis. The schematic of the loading condition is respectively shown.

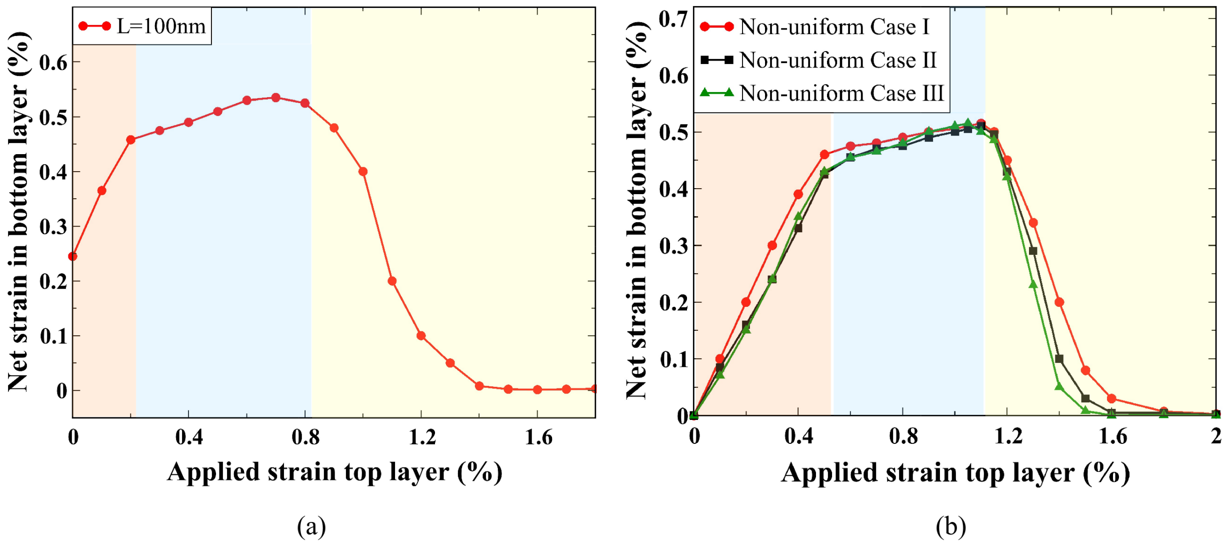

FIG. S8: Net strain in the bottom layer for (a) loading the fully unloaded configuration from 0.8% strain (Fig 5(a) of main text). (b) Non-uniform loading conditions-Case I: Uniform along x-axis and distributed load from flake center along y-axis (Fig. 4 main text), Case II: Uniform along x-axis and distributed load (0.8ϵ-1.2ϵ) along y-axis, Case III: Uniform along x-axis and distributed load (0.6ϵ-1.4ϵ) along y-axis (Fig. S5)

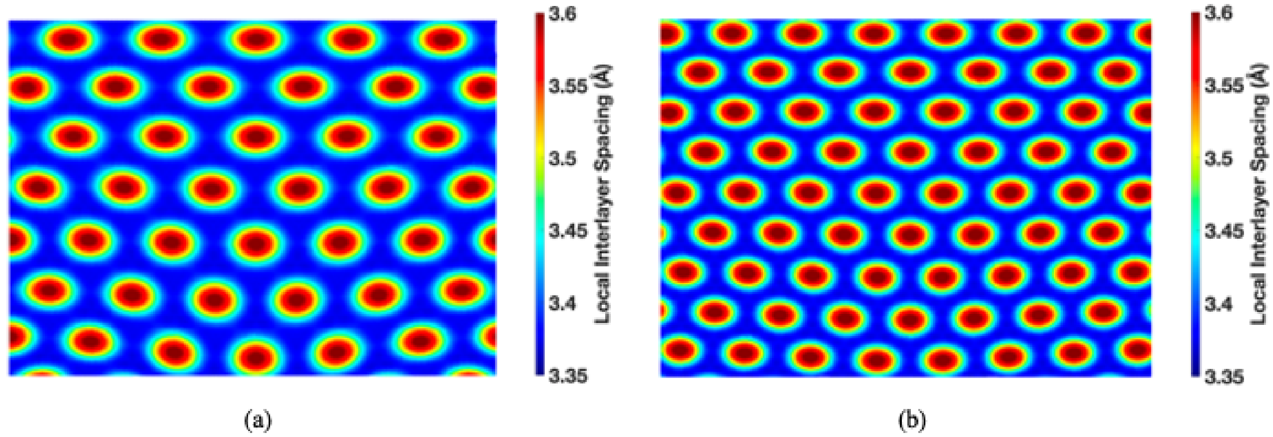

FIG. S9: Spatial variations of local interlayer spacing for biaxially heterostrained (tension) bilayer graphene 100 nm flake with (a) 1.4% and (b) 1.7% strain. The contour plot is based on the maximum and minimum interlayer spacing detected in the configurations.

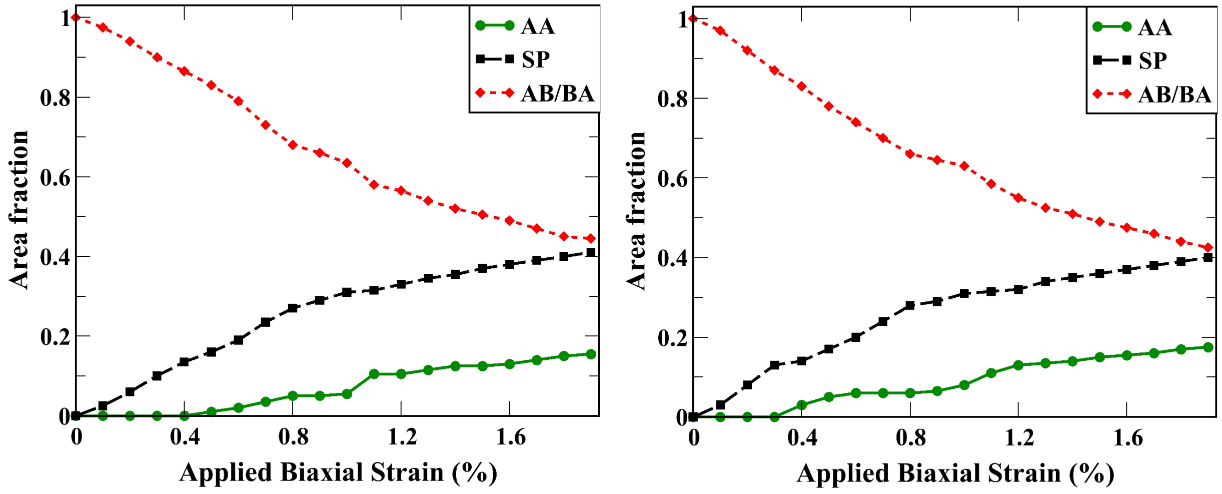

(a) (b)

FIG. S10: Sub-domain area fractions (AA, AB/BA, and SP stacking) of evolving moiré patterns as a function of applied biaxial strain to the top layer for (a) L = 100nm and (b) L = 250 nm flakes respectively. The analysis shows how the initially stacked AB-bilayer graphene shifts to a mix of local sub-domains with evolving SP and AA regions as strain is employed.

REFERENCES

1. Giannozzi, P., Baroni, S., Bonini, N., Calandra, M., Car, R., Cavazzoni, C., Ceresoli, D., Chiarotti, G. L., Cococcioni, M., Dabo, I., *et al.* QUANTUM ESPRESSO: a modular and open-source software project for quantum simulations of materials. *Journal of physics: Condensed matter* **21,** 395502 (2009).
2. Perdew, J. P., Burke, K. & Ernzerhof, M. Generalized Gradient Approximation Made Simple. *Phys. Rev. Lett.* **77,** 3865–3868 (18 1996).
3. Ernzerhof, M. & Scuseria, G. E. Assessment of the Perdew–Burke–Ernzerhof exchange-correlation functional. *The Journal of chemical physics* **110,** 5029–5036 (1999).
4. Sharma, R., Dey, A., Dar, S. A. & Srivastava, V. A DFT investigation of CsMgX3 (X= Cl, Br) halide perovskites: electronic, thermoelectric and optical properties. *Computational and Theoretical Chemistry* **1204,** 113415 (2021).
5. Dey, A., Sharma, R., Dar, S. A. & Wani, I. H. Cubic PbGeO3 perovskite oxide: a compound with striking electronic, thermoelectric and optical properties, explored using DFT studies. *Computational Condensed Matter* **26,** e00532 (2021).
6. Abraham, J. A., Sharma, R., Ahmad, S. & Dey, A. DFT investigation on the electronic, optical and thermoelectric properties of novel half-Heusler compounds ScAuX (X= Si, Ge, Sn, Pb) for energy harvesting technologies. *The European Physical Journal Plus* **136,** 1091 (2021).
7. Ehrlich, S., Moellmann, J. & Grimme, S. Dispersion-corrected density functional theory for aromatic interactions in complex systems. *Accounts of chemical research* **46,** 916–926 (2013).
8. Wisesa, P., McGill, K. A. & Mueller, T. Efficient generation of generalized Monkhorst-Pack grids through the use of informatics. *Physical Review B* **93,** 155109 (2016).
9. Wang, H., Zhang, L., Han, J. & Weinan, E. DeePMD-kit: A deep learning package for many-body potential energy representation and molecular dynamics. *Computer Physics Communications* **228,** 178–184 (2018).

10. Zhang, Y., Wang, H., Chen, W., Zeng, J., Zhang, L., Wang, H. & Weinan, E. DP-GEN: A concurrent learning platform for the generation of reliable deep learning based potential energy models. *Computer Physics Communications* **253,** 107206 (2020).

11. Zahradník, R. & Hobza, P. Weak intermolecular interactions: Statics and dynamics. *International Journal of Quantum Chemistry* **29,** 663–676 (1986).

12. Chowdhury, S. A., Inzani, K., Peña, T., Dey, A., Wu, S. M., Griffin, S. M. & Askari, H. Mechanical properties and strain transfer behavior of molybdenum ditelluride (MoTe2) thin films. *Journal of Engineering Materials and Technology* **144,** 011006 (2022).

13. Azizimanesh, A., Dey, A., Chowdhury, S. A., Wenner, E., Hou, W., Peña, T., Askari, H. & Wu, S. M. Strain engineering in 2D hBN and graphene with evaporated thin film stressors. *Applied Physics Letters* **123,** 043504 (July 2023).

14. Stukowski, A. Visualization and analysis of atomistic simulation data with OVITO–the Open Visualization Tool. *Modelling and simulation in materials science and engineering* **18,** 015012 (2009).

15. Zeng, J., Zhang, D., Lu, D., Mo, P., Li, Z., Chen, Y., Rynik, M., Huang, L., Li, Z., Shi, S., *et al.* DeePMD-kit v2: A software package for deep potential models. *The Journal of Chemical Physics* **159** (2023).

16. Nazareth, J. L. Conjugate gradient method. *Wiley Interdisciplinary Reviews: Computational Statistics* **1,** 348–353 (2009).
